# Mortality trends in the United States for adults with concurrent cerebrovascular disease and pulmonary embolism

**DOI:** 10.3389/fneur.2026.1738297

**Published:** 2026-02-26

**Authors:** Tian Lv, Yu-Jun Xiong, Yiqiao Chen

**Affiliations:** 1 Department of Neurology, Zhuji Affiliated Hospital of Wenzhou Medical University, Zhuji, China; 2 Department of Gastroenterology, Beijing Hospital, National Center of Gerontology, Institute of Geriatric Medicine, Chinese Academy of Medical Sciences, Beijing, China; 3 Department of Neurology, Qingtian People's Hospital, Lishui, China

**Keywords:** age-adjusted mortality rate, annual percent change, cerebrovascular disease, mortality trends, pulmonary embolism

## Abstract

**Background:**

Pulmonary embolism (PE) and cerebrovascular disease are major global causes of mortality and may share common risk factors. This study analyzed U.S. all-cause mortality trends where PE and cerebrovascular diseases were recorded on the death certificate from 1999 to 2023.

**Methods:**

Using national all-cause mortality data for adults aged over 25 years whose death certificates recorded both PE (ICD-10 I26) and cerebrovascular diseases (ICD-10 I60–I69), we calculated age-adjusted mortality rates (AAMRs), standardized to the 2000 U.S. population. Joinpoint regression was applied to identify significant trends and compute annual and average annual percent changes (APC and AAPC). Subgroup analyses were performed by sex, age, race, region, and urbanization level.

**Results:**

Between 1999 and 2023, 59,075 U.S. deaths involved both pulmonary embolism and cerebrovascular disease, with 4,274 recorded in 2023. Age-adjusted mortality increased from 1.00 to 1.55 per 100,000 (AAPC: 1.93%), accelerating sharply during 2018–2021. Higher AAMR was observed in males, adults over 85 years, Non-Hispanic Black individuals, residents of the South, and non-metropolitan areas. Substantial geographic heterogeneity existed, with states such as Minnesota, Washington, Massachusetts, and Florida showing significant long-term upward trends.

**Conclusion:**

The accelerating mortality and pronounced disparities across demographic and geographic groups highlight the need for more precise public health strategies. Mitigating this burden requires targeted interventions for high-risk populations, equity-focused policies, improved healthcare access, geriatric-sensitive care, and strengthened infrastructure in vulnerable regions.

## 1 Introduction

Pulmonary embolism and cerebrovascular diseases represent significant global health challenges. Pulmonary embolism, a critical manifestation of venous thromboembolism, causes approximately 60,000–100,000 annual deaths in the United States alone (1). Concurrently, cerebrovascular diseases remain a leading cause of mortality worldwide, accounting for approximately 7.3 million deaths in 2021 (2, 3). While traditionally considered distinct pathological entities, emerging epidemiological evidence suggests important clinical intersections between these conditions. Recent cohort studies have demonstrated that patients with ischemic stroke exhibit substantially elevated risks of subsequent venous thromboembolism, particularly within the first month post-event (4, 5).

While venous thromboembolism and cerebrovascular disease have conventionally been viewed as separate clinical entities, growing evidence points to interconnected pathophysiological pathways, common risk factors, and potential bidirectional relationships. The Tromsø Study, a large population-based cohort, demonstrated that patients experiencing an ischemic stroke faced a sharply increased risk of subsequent venous thromboembolism—particularly during the first month post-stroke (HR 19.7; 95% CI 10.1–38.5), with the hazard remaining elevated in the following months (6). This association was especially pronounced for pulmonary embolism (HR 20.2; 95% CI 7.4–55.1). Supporting this concept, prior studies have reported that in selected clinical settings, such as patients with patent foramen ovale, venous thromboembolism may be associated with an increased risk of subsequent ischemic stroke, highlighting a potential shared thromboembolic milieu (7). However, such mechanisms cannot be evaluated in death certificate–based data. Together, these findings highlight the need to examine pulmonary embolism and cerebrovascular disease not in isolation, but as jointly contributing to population mortality.

To address these knowledge gaps, we conducted a national population-based study using comprehensive mortality data from the CDC WONDER (1999–2023). Accordingly, the present study was designed as a descriptive, population-level analysis to characterize mortality trends in which pulmonary embolism and cerebrovascular disease were concurrently recorded on death certificates, rather than to infer causal relationships or temporal sequencing between events. Through detailed stratification by demographic and geographic factors, this study provides crucial insights into the evolving burden of these interconnected conditions and informs targeted public health interventions.

## 2 Materials and methods

### 2.1 Study design and inclusion criteria

This retrospective, population-based study used data from the CDC WONDER multiple cause-of-death data covering the period from 1999 to 2023, which include death certificate information for U.S. residents coded according to the International Classification of Diseases, Tenth Revision (ICD-10) (8). The analysis was based on de-identified, publicly accessible data from CDC WONDER, and thus ethical approval was not required. This study examined all-cause mortality among individuals aged over 25 years whose death certificates recorded both pulmonary embolism (ICD-10 I26) and cerebrovascular diseases (ICD-10 I60–I69) in the multiple-cause-of-death fields. Because CDC WONDER provides aggregated rather than individual-level records, it is not possible to enumerate stepwise exclusions or to quantify the number of deaths excluded due to missing demographic variables such as age, sex, or race. Consequently, exclusion counts by calendar year or subgroup and a conventional flow diagram of record selection could not be generated. This limitation reflects the structure of the data source rather than additional *post hoc* exclusions applied by the investigators (9).

### 2.2 Data extraction and subgroup analyses

Data on total deaths, population counts, and key demographic variables were obtained from the national database. Mortality rates were stratified by age group (25–34, 35–44, 45–54, 55–64, 65–74, 75–84 and 85 + years), sex, race (Hispanic, non-Hispanic White, non-Hispanic Black, and non-Hispanic Other), and U.S. Census region (Northeast, Midwest, South, and West). Urban–rural classification was based on the National Center for Health Statistics Urban–Rural Classification Scheme, with metropolitan areas encompassing large central, large fringe, medium, and small metropolitan counties, and nonmetropolitan areas including micropolitan and noncore counties (Supplementary Tables 1–4). This multilevel stratification enabled a comprehensive evaluation of mortality disparities across demographic, geographic, and urban–rural categories (10).

### 2.3 Statistical analysis

Crude mortality rates were computed as the yearly count of deaths with both pulmonary embolism and cerebrovascular diseases as contributing causes, divided by the corresponding U.S. population estimate for that year. Age-adjusted mortality rates (AAMRs) were derived using direct standardization based on the 2000 U.S. standard population. To evaluate temporal trends in AAMRs, we applied Joinpoint Regression Software (version 5.4.0.1; National Cancer Institute). This method identifies statistically significant inflection points by fitting multiple connected linear segments to the data. Joinpoint regression analyses were performed on log-transformed age-adjusted mortality rates (AAMRs), which is the standard approach for estimating temporal trends. For each segment, the annual percent change (APC) was calculated, and the average annual percent change (AAPC) over the full study period was derived as an overall summary measure. Statistical significance of APC and AAPC estimates was assessed using the internal t-test–based inference provided by the Joinpoint Regression Program (version 5.4.0.1, National Cancer Institute). Joinpoint regression fits log-linear models to age-adjusted mortality rates and estimates APCs for each temporal segment and AAPCs for the overall study period. The significance of these estimates is evaluated by the software using t-tests based on the standard errors of the estimated regression slopes. A two-sided *p* value < 0.05 was considered statistically significant. Together, these methods enabled a detailed and reliable analysis of national mortality trends within various demographic and geographic subgroups. It is important to note that mortality counts, AAMRs, and APC/AAPC estimates are presented as blanks or NA in Figure panels and Supplementary Tables 1–4 for specific states and years where data were insufficient or unavailable for calculation. These state–years with suppressed or sparse data were omitted entirely from the Joinpoint regression analyses and were not imputed. Given the large number of subgroup and state-level trend analyses, these results are intended to be descriptive and exploratory in nature. Accordingly, no formal adjustment for multiple comparisons was applied, and findings should be interpreted with appropriate caution.

## 3 Results

### 3.1 Overall characteristics

**Figure 1:**
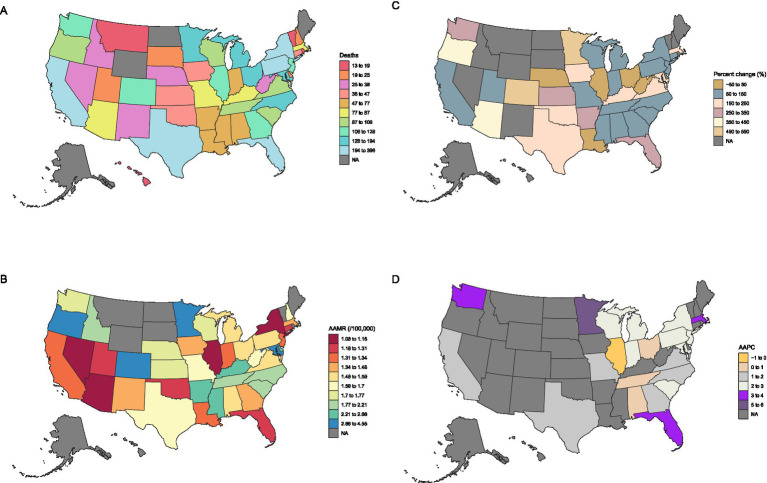
State-specific trends in all-cause mortality among patients with concurrent cerebrovascular disease and pulmonary embolism in the U.S. from 1999–2023: **(A)** Deaths; **(B)** AAMRs; **(C)** Percentage change; **(D)** AAPC. State–year observations with insufficient or suppressed data are shown as blanks or NA and were excluded from the Joinpoint regression analyses.

Between 1999 and 2023, a total of 59,075 deaths in the United States involved both conditions, including 4,274 deaths in 2023 alone—representing a 139.31% increase from the 1,786 deaths recorded in 1999 (Figures 1A–D and Table 1). Over this period, the overall AAMR increased from 1.00 (95% CI 0.95–1.04) to 1.55 (95% CI 1.50–1.60) per 100,000 population, corresponding to an AAPC of 1.93% (95% CI 0.91–2.97%, *p* < 0.05). Joinpoint regression analysis identified multiple inflection points in the temporal trend. From 1999 to 2013, the AAMR showed a modest but consistent decline, with an APC of −1.01% (95% CI − 1.41 to −0.61%). This was followed by a significant increase between 2013 and 2018 (APC 3.66, 95% CI 1.11–6.28%), and a marked acceleration from 2018 to 2021 (APC 18.57, 95% CI 11.03–26.61%, *p* < 0.05). In the most recent interval of 2021–2023, the mortality trend reversed direction, exhibiting a non-significant decline (APC − 4.40, 95% CI − 9.68 to 1.19%).

**Table 1:** Trends in mortality and age-adjusted mortality rates for concurrent cerebrovascular disease and pulmonary embolism in 1999 and 2023.

Characteristics	Deaths			AAMR		
	1999	2023	Percent change	1999	2023	AAPC (95% CI)
Overall	1786	4274	139.31	1.00 (0.95 to 1.04)	1.55 (1.50 to 1.60)	1.93 (0.91 to 2.97)*
Sex						
Female	1019	2247	120.51	0.96 (0.90 to 1.02)	1.48 (1.42 to 1.54)	1.91 (0.65 to 3.18)*
Male	767	2027	164.28	1.07 (0.99 to 1.15)	1.63 (1.56 to 1.71)	1.88 (0.43 to 3.36)*
Census region						
Northeast	303	678	123.76	0.80 (0.71 to 0.89)	1.30 (1.20 to 1.40)	2.20 (0.54 to 3.89)*
Midwest	475	905	90.53	1.13 (1.03 to 1.23)	1.57 (1.46 to 1.67)	1.63 (0.34 to 2.94)*
South	706	1729	144.9	1.12 (1.04 to 1.20)	1.66 (1.58 to 1.74)	1.75 (−0.10 to 3.62)
West	302	962	218.54	0.87 (0.77 to 0.96)	1.55 (1.45 to 1.65)	2.50 (0.96 to 4.07)*
Races						
Hispanic	56	287	412.5	0.63 (0.46 to 0.83)	0.98 (0.86 to 1.10)	1.95 (−1.35 to 5.36)
NH Black	353	853	141.64	2.35 (2.10 to 2.60)	3.11 (2.89 to 3.32)	1.44 (−0.34 to 3.25)
NH White	1355	2978	119.78	0.93 (0.88 to 0.98)	1.49 (1.43 to 1.54)	2.15 (0.98 to 3.34)*
NH Other	20	139	595	0.42 (0.25 to 0.66)	0.72 (0.60 to 0.84)	NA
Urbanization						
Metropolitan	1412	3093	119.05	0.98 (0.92 to 1.03)	1.38 (1.33 to 1.43)	1.60 (0.77 to 2.45)*
Nonmetropolitan	374	649	73.53	1.11 (1.00 to 1.22)	1.49 (1.37 to 1.61)	1.46 (0.49 to 2.43)*
Age groups						
25–34 years		34	NA	NA	0.07 (0.05 to 0.10)	NA
35–44 years	42	98	133.33	0.09 (0.07 to 0.13)	0.22 (0.18 to 0.27)	3.82 (1.83 to 5.84)*
45–54 years	75	211	181.33	0.21 (0.16 to 0.26)	0.52 (0.45 to 0.59)	3.33 (0.37 to 6.39)*
55–64 years	200	653	226.5	0.84 (0.72 to 0.96)	1.56 (1.44 to 1.68)	2.72 (1.02 to 4.46)*
65–74 years	392	1106	182.14	2.13 (1.92 to 2.34)	3.19 (3.00 to 3.38)	1.72 (0.42 to 3.04)*
75–84 years	641	1244	94.07	5.24 (4.84 to 5.65)	6.77 (6.40 to 7.15)	1.51 (0.63 to 2.40)*
85 + years	427	928	117.33	10.28 (9.30 to 11.25)	14.98 (14.02 to 15.94)	1.96 (0.53 to 3.42)*

* indicates statistically significant AAPC.

#Urbanization-specific AAMR for 2023 is based on 2020 data. AAPC was calculated for 1999–2023.

##Age-group AAMR and APC are calculated using crude rates.

NA means no data uploaded about this field.

AAMR, Age adjusted mortality rate; CI, confidence interval; AAPC, average annual percentage change, NH, non-Hispanic.

### 3.2 Sex-stratified analyses

**Figure 2:**
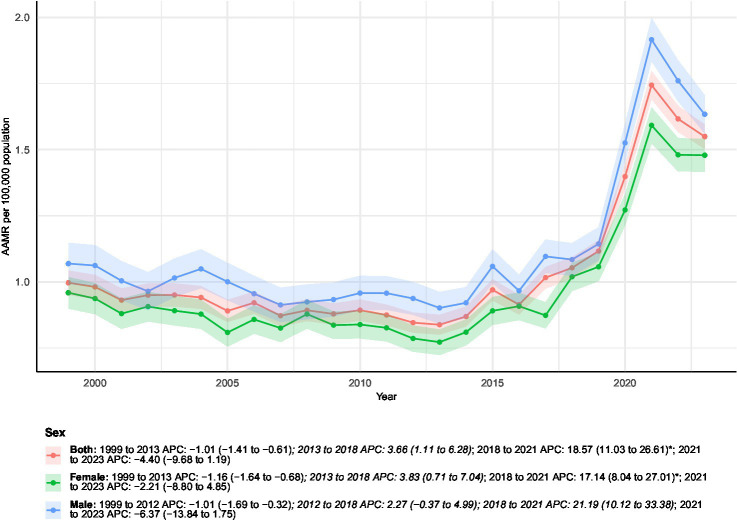
Age-adjusted mortality rates (AAMRs) of all-cause mortality among patients with concurrent cerebrovascular disease and pulmonary embolism stratified by sex from 1999–2023. Blank or NA values represent years with insufficient data and were excluded from the Joinpoint analyses.

Among individuals with both conditions, a total of 27,442 male and 31,633 female deaths were recorded between 1999 and 2023. Female deaths increased from 1,019 in 1999 to 2,247 in 2023, representing a 120.51% rise (Figure 2). The AAMR for females increased from 0.96 (95% CI 0.90–1.02) to 1.48 (95% CI 1.42–1.54) per 100,000 population, with an overall AAPC of 1.91% (95% CI 0.65–3.18%, *p* < 0.05). Joinpoint regression showed an initial decline from 1999 to 2013 (APC − 1.16, 95% CI − 1.64 to −0.68%), followed by significant increases during 2013–2018 (APC 3.83, 95% CI 0.71–7.04%) and 2018–2021 (APC 17.14, 95% CI 8.04–27.01%, *p* < 0.05). From 2021 to 2023, the trend reversed toward a nonsignificant decrease (APC − 2.21, 95% CI − 8.80 to 4.85%).

Among males, deaths increased from 767 in 1999 to 2,027 in 2023, reflecting a 164.28% rise. The male AAMR rose from 1.07 (95% CI 0.99–1.15) to 1.63 (95% CI 1.56–1.71) per 100,000 population, corresponding to an AAPC of 1.88% (95% CI 0.43–3.36%, *p* < 0.05). Trend analysis identified a moderate decline between 1999 and 2012 (APC − 1.01, 95% CI − 1.69 to −0.32%), followed by an upward shift during 2012–2018 (APC 2.27, 95% CI − 0.37 to 4.99%) and a sharp surge from 2018 to 2021 (APC 21.19, 95% CI 10.12–33.38%, *p* < 0.05). A nonsignificant decline was observed from 2021 to 2023 (APC − 6.37, 95% CI − 13.84 to 1.75%).

### 3.3 Age-stratified analyses

**Figure 3:**
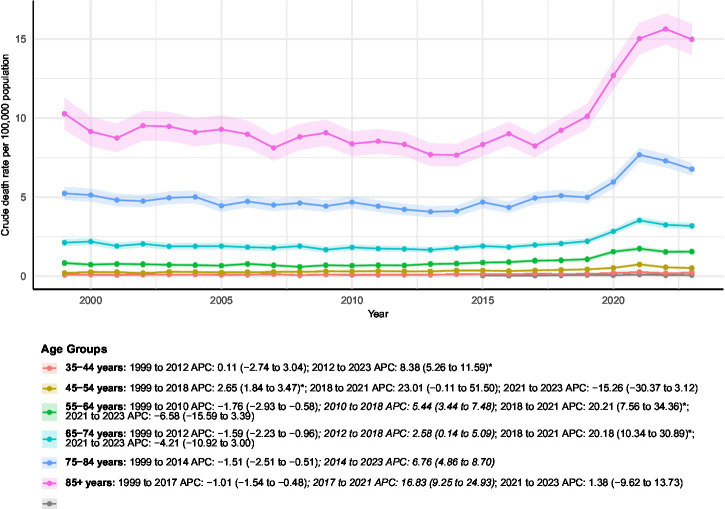
AAMRs of all-cause mortality among patients with concurrent cerebrovascular disease and pulmonary embolism stratified by age group from 1999–2023. Blank or NA values represent years with insufficient data and were excluded from the Joinpoint analyses.

Age-stratified analyses demonstrated substantial heterogeneity in mortality patterns between 1999 and 2023 (Figure 3), with clear distinctions between absolute mortality burden and relative temporal increases across age groups. For subgroups with sparse data and unavailable age-adjusted mortality rates, we refrained from interpreting relative changes in crude death counts or inferring temporal trends.

Among adults aged 25–34 years, the absolute number of deaths remained very small throughout the study period, and age-adjusted mortality rates could not be reliably estimated due to sparse data. Accordingly, no temporal trend was inferred for this age group. In the 35–44-year group, a total of 1,353 deaths occurred during the study period, with annual deaths increasing from 42 to 98 (a 133.33% increase). The AAMR rose from 0.09 (95% CI 0.07–0.13) to 0.22 (95% CI 0.18–0.27), corresponding to an AAPC of 3.82% (95% CI 1.83–5.84%, *p* < 0.05). Joinpoint analysis indicated a stable early phase from 1999–2012 (APC 0.11, 95% CI − 2.74 to 3.04%), followed by a statistically significant acceleration during 2012–2023 (APC 8.38, 95% CI 5.26–11.59%, *p* < 0.05).

Adults aged 45–54 years experienced 3,696 total deaths, with the AAMR increasing from 0.21 (95% CI 0.16–0.26) to 0.52 (95% CI 0.45–0.59) and an AAPC of 3.33% (95% CI 0.37–6.39%, *p* < 0.05). Mortality rose significantly during 1999–2018 (APC 2.65, 95% CI 1.84–3.47%, *p* < 0.05), followed by a sharp but nonsignificant increase in 2018–2021 and a nonsignificant decline thereafter.

Middle-aged and younger-old adults also demonstrated notable relative increases. The 55–64-year group accounted for 8,488 cumulative deaths, with AAMR rising from 0.84 to 1.56 and an AAPC of 2.72% (95% CI 1.02–4.46%, *p* < 0.05). Trends included a significant decline from 1999–2010 (APC − 1.76, 95% CI − 2.93 to −0.58%, *p* < 0.05), followed by a significant increase in 2010–2018 (APC 5.44, 95% CI 3.44–7.48%, *p* < 0.05) and a pronounced surge during 2018–2021 (APC 20.21, 95% CI 7.56–34.36%, *p* < 0.05). In the 65–74-year group, 13,450 deaths were recorded, and the AAMR increased from 2.13 to 3.19, with an AAPC of 1.72% (95% CI 0.42–3.04%, *p* < 0.05). After an initial decline from 1999–2012 (APC − 1.59, 95% CI − 2.23 to −0.96%, *p* < 0.05), mortality rose moderately during 2012–2018 and then sharply during 2018–2021 (APC 20.18, 95% CI 10.34–30.89%, *p* < 0.05).

In contrast, adults aged 85 years and older consistently exhibited the highest mortality levels of all age groups, representing the greatest absolute burden across the age spectrum. This group accounted for 13,639 cumulative deaths, and the AAMR increased from 10.28 (95% CI 9.30–11.25) to 14.98 (95% CI 14.02–15.94). Joinpoint analysis showed a significant decline during 1999–2017 (APC − 1.01, 95% CI − 1.54 to −0.48%, *p* < 0.05), followed by a marked surge during 2017–2021 (APC 16.83, 95% CI 9.25–24.93%, *p* < 0.05) and a nonsignificant change from 2021–2023. Despite steeper relative increases observed in several younger age groups, absolute AAMRs remained substantially higher among the oldest adults throughout the study period.

### 3.4 Regional-stratified analyses

#### 3.4.1 Census regions stratified

**Figure 4:**
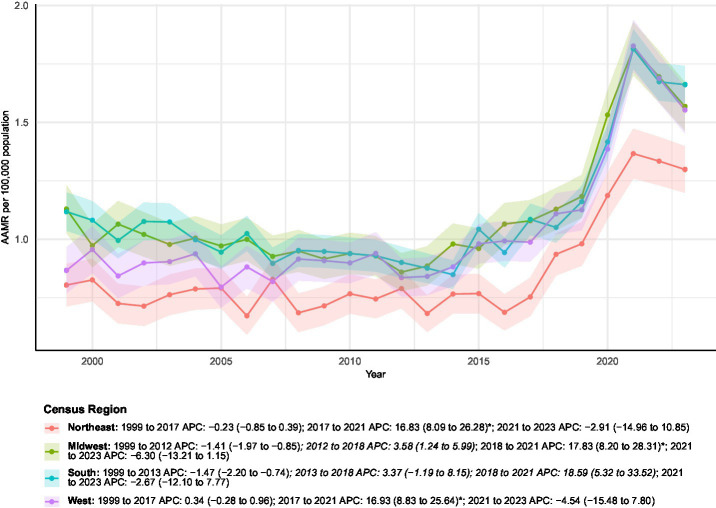
AAMRs of all-cause mortality among patients with concurrent cerebrovascular disease and pulmonary embolism stratified by census region from 1999–2023. Blank or NA values represent years with insufficient data and were excluded from the Joinpoint analyses.

Regional analyses revealed marked geographic variability in mortality burden across the four U.S. Census regions (Table 1 and Figure 4). The South accounted for the largest number of deaths (*n* = 22,907), followed by the Midwest (*n* = 13,908), West (*n* = 12,781), and Northeast (*n* = 9,479). Annual deaths increased in all regions, with the most pronounced relative rise in the West (from 302 to 962 deaths; +218.5%). The West also demonstrated a steep increase in AAMR, from 0.87 to 1.55 (AAPC 2.50%; *p* < 0.05), characterized by stability from 1999–2017 followed by a sharp, significant rise during 2017–2021.

In the Northeast, the AAMR increased from 0.80 to 1.30 (AAPC 2.20%; *p* < 0.05), with a similar pattern of long-term stability (1999–2017) preceding a significant surge in 2017–2021. The Midwest showed an AAMR increase from 1.13 to 1.57 (AAPC 1.63%; *p* < 0.05), with trends indicating an early decline (1999–2012), followed by increasing mortality and a marked, significant rise during 2018–2021. Despite bearing the greatest overall mortality burden and a substantial rise in annual deaths (from 706 to 1,729), the South’s AAMR increase from 1.12 to 1.66 corresponded to a non-significant AAPC of 1.75%. Its trend was marked by an early significant decline (1999–2013) followed by subsequent non-significant increases, including a pronounced surge in 2018–2021.

#### 3.4.2 State-stratified

Substantial heterogeneity in mortality trends was observed across U.S. states. Significant long-term increases in age-adjusted mortality were documented in several jurisdictions. Florida experienced one of the most pronounced rises (AAPC 3.27, 95% CI 1.73–4.82%). Other states with significant upward trends included Minnesota (AAPC 5.34, 95% CI 3.40–7.31%), Washington (AAPC 3.14, 95% CI 0.19–6.18%), Massachusetts (AAPC 3.10, 95% CI 0.62–5.64%), Michigan (AAPC 2.95, 95% CI 1.36–4.58%), New Jersey (AAPC 2.63, 95% CI 0.67–4.62%), Wisconsin (AAPC 2.58, 95% CI 0.40–4.81%), Indiana (AAPC 2.54, 95% CI 1.40–3.68%), New York (AAPC 2.24, 95% CI 0.36–4.16%), South Carolina (AAPC 2.21, 95% CI 0.32–4.14%), Texas (AAPC 1.96, 95% CI 0.99–2.95%), and California (AAPC 1.54, 95% CI 0.50–2.60%). These states demonstrated consistent upward trajectories throughout the study period.

For many other states, including Alaska, Delaware, Hawaii, Idaho, Kansas, Maine, Montana, Nebraska, Nevada, New Hampshire, New Mexico, North Dakota, Oklahoma, Rhode Island, South Dakota, Utah, Vermont, and Wyoming, long-term trends could not be reliably assessed due to incomplete data or unavailable AAPC estimates for the study period.

### 3.5 Race-stratified analyses

**Figure 5:**
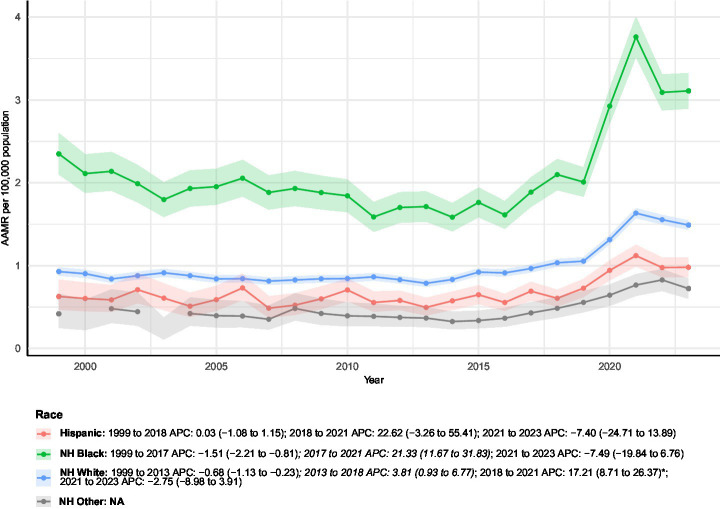
AAMRs of all-cause mortality among patients with concurrent cerebrovascular disease and pulmonary embolism stratified by race from 1999–2023. Blank or NA values represent years with insufficient data and were excluded from the Joinpoint analyses.

Substantial differences in mortality burden were observed across racial groups (Figure 5). Non-Hispanic White individuals accounted for the most deaths (*n* = 43,346) and were the only group with a statistically significant long-term increase in age-adjusted mortality (AAPC 2.15%). Their trend included a pronounced surge from 2018 to 2021.

While Non-Hispanic Black individuals experienced a 141.6% increase in crude mortality, the long-term AAPC was not statistically significant. Their mortality pattern showed an initial decline, a sharp but transient rise from 2017–2021, and a subsequent decrease.

Among Hispanic individuals, crude mortality rose by 412.5%, but no significant long-term trend in AAMR was observed. Their pattern featured prolonged stability, a brief spike (2018–2021), and a subsequent reversal.

The Non-Hispanic Other group showed the largest relative increase in crude deaths (595%), but unavailable AAPC and Joinpoint data precluded trend interpretation for this population.

### 3.6 Urbanization-stratified analyses

**Figure 6:**
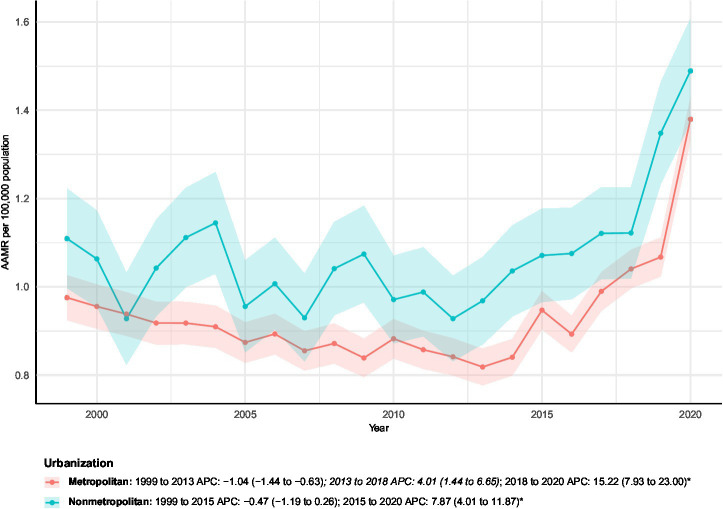
AAMRs of all-cause mortality among patients with concurrent cerebrovascular disease and pulmonary embolism stratified by urbanization from 1999–2023. Blank or NA values represent years with insufficient data and were excluded from the Joinpoint analyses.

Mortality patterns varied by urbanization level (Figure 6). Both urbanization categories exhibited statistically significant long-term increases in age-adjusted mortality: metropolitan areas showed an AAPC of 1.60%, and nonmetropolitan areas had an AAPC of 1.46%.

Joinpoint analyses revealed distinct temporal patterns. In metropolitan regions, mortality rate declined from 1999 to 2013, followed by moderate growth from 2013 to 2018, and then a sharp, statistically significant increase during 2018–2020. Nonmetropolitan areas exhibited a smaller early decline (1999–2015), followed by a significant rise from 2015 to 2020. This pattern indicates a more recent acceleration in mortality in nonmetropolitan areas compared to metropolitan regions.

## 4 Discussion

This study is designed as a descriptive, population-level surveillance analysis of mortality trends involving pulmonary embolism and cerebrovascular disease, rather than an inferential or causal investigation. Our national mortality analysis reveals a notable shift in age-adjusted mortality rates for deaths involving both pulmonary embolism and cerebrovascular diseases among U.S. adults. Following a substantial decline from 1999 to 2011, mortality rates demonstrated a modest but statistically significant increase from 2011 to 2023. Throughout this period, persistent disparities were observed, with consistently higher mortality among males, adults aged 85 years and older, non-Hispanic Black individuals, residents of the U.S. South, and those in nonmetropolitan areas.

The pronounced acceleration in mortality observed during 2018–2021 coincides temporally with the COVID-19 pandemic and warrants careful interpretation. Accumulating evidence indicates that SARS-CoV-2 infection is strongly associated with systemic inflammation, endothelial dysfunction, and hypercoagulability (11), substantially increasing the risk of venous thromboembolism, including pulmonary embolism, as well as acute cerebrovascular events (12, 13). In addition to direct infection-related thrombosis, indirect pandemic-related factors may have contributed to the observed mortality surge (14). These include disruptions in healthcare access, delayed emergency presentation, reduced utilization of preventive and follow-up care, and strained hospital resources, all of which disproportionately affected older adults and socioeconomically vulnerable populations. Changes in death certification practices during the pandemic period may also have influenced cause-of-death reporting, with greater attention to thrombotic complications and multi-morbidity in critically ill patients (15).

The initial decline likely reflects advancements in prevention, diagnosis, and treatment for both conditions. Improved hypertension control, dyslipidemia management, and smoking reduction have contributed to decreased cerebrovascular mortality (16), while enhanced imaging capabilities and optimized anticoagulation protocols have improved pulmonary embolism outcomes (17). However, the subsequent reversal after approximately 2011 suggests the emergence of countervailing factors, including population aging, rising obesity prevalence, and persistent healthcare disparities. This pattern aligns with recent studies showing increasing crude stroke mortality numbers despite continued age-standardized rate declines, particularly concerning are the emerging upticks in younger age groups (18).

The persistent male–female mortality disparity warrants multifaceted interpretation. Men typically exhibit higher prevalence of cardiovascular risk factors and historically demonstrate elevated rates of both venous thromboembolism and stroke (19, 20). Biological differences in hemostatic mechanisms and endothelial function may contribute, though evidence remains limited regarding their role in the co-occurrence of these conditions. From a healthcare delivery perspective, delayed presentation and reduced engagement with preventive services among men may further exacerbate this disparity, highlighting the need for sex-specific prevention strategies (21).

The striking mortality burden among adults aged 85 years and older reflects the compounding effects of multimorbidity, reduced physiological reserve, and functional limitations. Older adults often face diagnostic challenges, treatment limitations, and reduced access to specialized care, particularly in rural settings (22). These findings emphasize the necessity of developing geriatric-adapted clinical pathways that incorporate comprehensive assessment and frailty evaluation to optimize prevention and management strategies for this vulnerable population.

Geographic disparities, particularly the elevated mortality in the U.S. South, echo the well-documented “Stroke Belt” phenomenon (23). This pattern likely stems from the interplay of multiple factors, including higher prevalence of cardiovascular risk factors, socioeconomic challenges, and limited healthcare access (24). Rural areas within these regions face additional barriers, including longer transport times, fewer specialist providers, and reduced availability of advanced imaging capabilities (25). Addressing these disparities requires targeted investments in telehealth infrastructure, mobile health units, and regionalized systems of care to ensure equitable access to evidence-based interventions.

The consistently elevated mortality among non-Hispanic Black individuals underscores the profound impact of health inequities. While biological differences have been hypothesized, substantial evidence indicates that social determinants of health, structural barriers to care, and differential treatment quality represent primary drivers of this disparity (26). Achieving health equity will require multifaceted approaches addressing insurance coverage gaps, improving cultural competence in healthcare delivery, and implementing community-based prevention programs tailored to underserved populations.

The rural–urban mortality divide further emphasizes the critical role of healthcare infrastructure. Rural residents face numerous barriers, including limited specialist availability, delayed diagnostic capabilities, and prolonged transfer times for definitive care (27, 28). Strengthening rural healthcare systems through telehealth expansion, hub-and-spoke care models, and workforce development initiatives represents an essential component of comprehensive mortality reduction strategies.

Moving forward, effective interventions should prioritize high-risk populations through targeted screening and prevention programs. Healthcare systems must develop tailored approaches for rural and southern communities, including infrastructure investments and care model innovations. For older adults, integrating geriatric assessment into routine care may improve risk stratification and management. Furthermore, addressing social determinants of health through cross-sector collaboration remains fundamental to achieving sustainable health equity. Continued surveillance and research into the pathophysiological interplay between pulmonary embolism and cerebrovascular diseases will be essential to guide future innovation in prevention and treatment strategies.

Our findings, while revealing significant mortality patterns, should be interpreted in light of several methodological limitations inherent to this type of national database analysis. Identification of pulmonary embolism and cerebrovascular diseases relied on their documentation on the death certificate and does not necessarily indicate acute events, temporal co-occurrence, or direct contribution to death. Cerebrovascular disease may reflect acute, chronic, or historical conditions, which cannot be distinguished using CDC WONDER data. Additionally, the absence of detailed clinical variables, including specific diagnostic methods, treatment regimens, and timing of events, precludes sensitivity analyses restricted to deaths in which either condition was the underlying cause with the other as a contributing cause. Additionally, as an ecological analysis based on aggregated mortality data, this study cannot assess individual-level associations, interactions, or causal pathways. The observed trends should therefore be interpreted as descriptive and hypothesis-generating. Importantly, the temporal sequence between cerebrovascular disease and pulmonary embolism cannot be determined using death certificate–based data, and thus distinct clinical scenarios (e.g., post-stroke venous thrombosis versus paradoxical embolism) cannot be differentiated. Future studies incorporating comprehensive electronic health record data—including imaging results, anticoagulation management, and neurological status—are needed to elucidate the underlying biological and clinical pathways linking these conditions. Future research should first seek to identify the specific mechanisms that might explain this association at the individual level, and then evaluate whether interventions derived from this understanding can effectively reduce mortality risk in patients with both conditions.

## 5 Conclusion

Our study reveals a significant and accelerating U.S. mortality burden from co-occurring pulmonary embolism and cerebrovascular disease, with a critical inflection point during 2018–2021. Mortality trends demonstrated substantial heterogeneity, identifying high-risk populations including older adults aged over 85, Non-Hispanic Black individuals, and residents of specific geographic regions. The observed synergistic trend between these conditions at the population level suggests that a siloed approach to disease management may be suboptimal. Future mitigation requires targeted, integrated strategies—such as enhanced risk stratification, optimized antithrombotic management, and improved care coordination—tailored to the vulnerabilities of these distinct populations.

## Data Availability

The datasets presented in this study can be found in online repositories. The names of the repository/repositories and accession number(s) can be found in the article/Supplementary material.
