# Supplementary material for: Mortality trends in the United States for adults with concurrent cerebrovascular disease and pulmonary embolism
Source: Front Neurol. 2026 Feb 26;17:1738297. doi: 10.3389/fneur.2026.1738297 (PMC12979155; doi:10.3389/fneur.2026.1738297)
Supplement: Supplementary file 1 [file Table_1.DOCX]

Supplementary table 1. State-specific Mortality and Age-Adjusted Mortality Rates for Concurrent Cerebrovascular Disease and Pulmonary Embolism in 1999 and 2023.

| States | Deaths | | | AAMR | | |
| --- | --- | --- | --- | --- | --- | --- |
|  | 1999 | 2023 | Percent Change | 1999 | 2023 | AAPC (95% CI) |
| Alabama | 38 | 65 | 71.05 | 1.30 (0.92 to 1.79) | 1.57 (1.20 to 2.01) | 0.14 (-1.91 to 2.24) |
| Alaska |  |  | NA | NA (NA to NA) | NA (NA to NA) |  |
| Arizona | 17 | 77 | 352.94 | NA (0.30 to 0.83) | 1.12 (0.88 to 1.40) |  |
| Arkansas | 16 | 56 | 250 | NA (0.50 to 1.43) | 2.24 (1.67 to 2.92) |  |
| California | 169 | 398 | 135.5 | 0.92 (0.78 to 1.05) | 1.31 (1.18 to 1.44) | 1.54 (0.50 to 2.60)* |
| Colorado | 21 | 122 | 480.95 | 1.00 (0.62 to 1.53) | 2.88 (2.36 to 3.39) |  |
| Connecticut | 17 | 40 | 135.29 | NA (0.41 to 1.12) | 1.22 (0.87 to 1.67) |  |
| Delaware |  | 22 | NA | NA (NA to NA) | 2.58 (1.55 to 4.02) |  |
| District of Columbia | 16 | 21 | 31.25 | NA (2.54 to 7.23) | 4.55 (2.82 to 6.95) |  |
| Florida | 67 | 272 | 305.97 | 0.49 (0.38 to 0.63) | 1.18 (1.03 to 1.32) | 3.27 (1.73 to 4.82)* |
| Georgia | 52 | 109 | 109.62 | 1.29 (0.96 to 1.69) | 1.39 (1.13 to 1.66) | 1.49 (-0.42 to 3.44) |
| Hawaii |  | 16 | NA | NA (NA to NA) | NA (0.64 to 1.90) |  |
| Idaho |  | 31 | NA | NA (NA to NA) | 2.12 (1.43 to 3.03) |  |
| Illinois | 115 | 121 | 5.22 | 1.51 (1.23 to 1.79) | 1.15 (0.94 to 1.36) | -0.19 (-1.57 to 1.21) |
| Indiana | 41 | 72 | 75.61 | 1.08 (0.78 to 1.47) | 1.31 (1.02 to 1.65) | 2.54 (1.40 to 3.68)* |
| Iowa | 14 | 42 | 200 | NA (0.30 to 0.93) | 1.44 (1.03 to 1.95) |  |
| Kansas | 10 | 42 | 320 | NA (0.27 to 1.02) | 1.72 (1.23 to 2.33) |  |
| Kentucky | 28 | 83 | 196.43 | 1.12 (0.75 to 1.63) | 2.21 (1.75 to 2.76) |  |
| Louisiana | 41 | 47 | 14.63 | 1.57 (1.13 to 2.13) | 1.32 (0.96 to 1.76) |  |
| Maine | 13 |  | NA | NA (0.76 to 2.43) | NA (NA to NA) |  |
| Maryland | 44 | 142 | 222.73 | 1.46 (1.06 to 1.96) | 2.79 (2.32 to 3.25) | 2.63 (-2.04 to 7.53) |
| Massachusetts | 26 | 82 | 215.38 | 0.59 (0.39 to 0.86) | 1.34 (1.06 to 1.67) | 3.10 (0.62 to 5.64)* |
| Michigan | 59 | 130 | 120.34 | 0.96 (0.73 to 1.24) | 1.48 (1.22 to 1.74) | 2.95 (1.36 to 4.58)* |
| Minnesota | 21 | 128 | 509.52 | 0.69 (0.43 to 1.05) | 2.66 (2.19 to 3.13) | 5.34 (3.40 to 7.31)* |
| Mississippi | 28 | 57 | 103.57 | 1.60 (1.06 to 2.31) | 2.43 (1.83 to 3.17) |  |
| Missouri | 50 | 82 | 64 | 1.29 (0.96 to 1.70) | 1.61 (1.27 to 2.01) | 1.27 (-0.21 to 2.77) |
| Montana |  | 17 | NA | NA (NA to NA) | NA (0.96 to 2.72) |  |
| Nebraska | 18 | 26 | 44.44 | NA (0.86 to 2.30) | 1.75 (1.14 to 2.59) |  |
| Nevada |  | 29 | NA | NA (NA to NA) | 1.08 (0.72 to 1.57) |  |
| New Hampshire |  | 23 | NA | NA (NA to NA) | 1.68 (1.04 to 2.56) |  |
| New Jersey | 44 | 106 | 140.91 | 0.78 (0.57 to 1.05) | 1.33 (1.08 to 1.59) | 2.63 (0.67 to 4.62)* |
| New Mexico |  | 25 | NA | NA (NA to NA) | 1.36 (0.87 to 2.03) |  |
| New York | 82 | 199 | 142.68 | 0.66 (0.53 to 0.83) | 1.12 (0.96 to 1.28) | 2.24 (0.36 to 4.16)* |
| North Carolina | 85 | 165 | 94.12 | 1.75 (1.39 to 2.16) | 1.84 (1.55 to 2.13) | 1.42 (-0.54 to 3.41) |
| North Dakota |  |  | NA | NA (NA to NA) | NA (NA to NA) |  |
| Ohio | 104 | 149 | 43.27 | 1.36 (1.10 to 1.62) | 1.49 (1.24 to 1.74) | 0.31 (-3.54 to 4.31) |
| Oklahoma | 14 | 38 | 171.43 | NA (0.33 to 1.01) | 1.21 (0.85 to 1.66) |  |
| Oregon | 19 | 99 | 421.05 | NA (0.50 to 1.31) | 2.81 (2.27 to 3.43) |  |
| Pennsylvania | 105 | 194 | 84.76 | 1.09 (0.88 to 1.30) | 1.58 (1.35 to 1.80) | 2.12 (-1.09 to 5.43) |
| Rhode Island |  | 16 | NA | NA (NA to NA) | NA (0.90 to 2.55) |  |
| South Carolina | 40 | 87 | 117.5 | 1.69 (1.21 to 2.30) | 1.93 (1.53 to 2.39) | 2.21 (0.32 to 4.14)* |
| South Dakota |  | 19 | NA | NA (NA to NA) | NA (1.58 to 4.23) |  |
| Tennessee | 52 | 100 | 92.31 | 1.48 (1.10 to 1.94) | 1.77 (1.41 to 2.12) | 0.61 (-3.14 to 4.51) |
| Texas | 120 | 332 | 176.67 | 1.14 (0.93 to 1.34) | 1.68 (1.50 to 1.87) | 1.96 (0.99 to 2.95)* |
| Utah | 11 | 23 | 109.09 | NA (0.57 to 2.03) | 1.20 (0.75 to 1.82) |  |
| Vermont |  | 13 | NA | NA (NA to NA) | NA (1.06 to 3.41) |  |
| Virginia | 36 | 104 | 188.89 | 0.92 (0.64 to 1.27) | 1.49 (1.20 to 1.78) | 1.14 (-0.72 to 3.03) |
| Washington | 27 | 109 | 303.7 | 0.79 (0.52 to 1.15) | 1.76 (1.43 to 2.10) | 3.14 (0.19 to 6.18)* |
| West Virginia | 21 | 29 | 38.1 | 1.54 (0.95 to 2.35) | 1.63 (1.08 to 2.36) |  |
| Wisconsin | 37 | 87 | 135.14 | 1.00 (0.70 to 1.38) | 1.70 (1.35 to 2.11) | 2.58 (0.40 to 4.81)* |
| Wyoming |  |  | NA | NA (NA to NA) | NA (NA to NA) |  |

* indicates statistically significant AAPC.

#Age-group AAMR and APC are calculated using crude rates.

NA means no data uploaded about this field.

Abbreviations: Age adjusted mortality rate (AAMR), confidence interval (CI), average annual percentage change (AAPC), non-Hispanic (NH).

Supplementary table 2. Age-Adjusted Mortality Rates and Standard Errors by Characteristics Across Years.

| Characteristics | Year | AAMR | SE |
| --- | --- | --- | --- |
| Census Region_Midwest | 1999 | 1.128517032 | 0.052008247 |
| Census Region_Midwest | 2000 | 0.972692002 | 0.047667898 |
| Census Region_Midwest | 2001 | 1.064593605 | 0.050146328 |
| Census Region_Midwest | 2002 | 1.020559558 | 0.048842175 |
| Census Region_Midwest | 2003 | 0.977557812 | 0.04717607 |
| Census Region_Midwest | 2004 | 1.004742386 | 0.047561715 |
| Census Region_Midwest | 2005 | 0.971141701 | 0.046769489 |
| Census Region_Midwest | 2006 | 0.999333495 | 0.047089932 |
| Census Region_Midwest | 2007 | 0.926214329 | 0.045057384 |
| Census Region_Midwest | 2008 | 0.949575306 | 0.045696099 |
| Census Region_Midwest | 2009 | 0.915549075 | 0.043836966 |
| Census Region_Midwest | 2010 | 0.938907544 | 0.044990493 |
| Census Region_Midwest | 2011 | 0.928375397 | 0.044023601 |
| Census Region_Midwest | 2012 | 0.859027566 | 0.041701914 |
| Census Region_Midwest | 2013 | 0.88527883 | 0.04280413 |
| Census Region_Midwest | 2014 | 0.979530939 | 0.044457275 |
| Census Region_Midwest | 2015 | 0.959934824 | 0.043853456 |
| Census Region_Midwest | 2016 | 1.064828151 | 0.045700476 |
| Census Region_Midwest | 2017 | 1.078777425 | 0.04520095 |
| Census Region_Midwest | 2018 | 1.128580408 | 0.04555785 |
| Census Region_Midwest | 2019 | 1.183216481 | 0.046728348 |
| Census Region_Midwest | 2020 | 1.532088849 | 0.053551243 |
| Census Region_Midwest | 2021 | 1.816150892 | 0.058132466 |
| Census Region_Midwest | 2022 | 1.695504936 | 0.055710788 |
| Census Region_Midwest | 2023 | 1.568317475 | 0.053515728 |
| Census Region_Northeast | 1999 | 0.803797775 | 0.046507578 |
| Census Region_Northeast | 2000 | 0.825573458 | 0.046661391 |
| Census Region_Northeast | 2001 | 0.724879063 | 0.043314682 |
| Census Region_Northeast | 2002 | 0.713527969 | 0.043007798 |
| Census Region_Northeast | 2003 | 0.762373592 | 0.044372036 |
| Census Region_Northeast | 2004 | 0.786901149 | 0.045056501 |
| Census Region_Northeast | 2005 | 0.790631693 | 0.044399118 |
| Census Region_Northeast | 2006 | 0.671923105 | 0.040868756 |
| Census Region_Northeast | 2007 | 0.828703365 | 0.046077865 |
| Census Region_Northeast | 2008 | 0.684951678 | 0.041972281 |
| Census Region_Northeast | 2009 | 0.714595124 | 0.041828501 |
| Census Region_Northeast | 2010 | 0.766371086 | 0.043100793 |
| Census Region_Northeast | 2011 | 0.744254609 | 0.042036195 |
| Census Region_Northeast | 2012 | 0.788648802 | 0.042611753 |
| Census Region_Northeast | 2013 | 0.682043585 | 0.040135743 |
| Census Region_Northeast | 2014 | 0.765426811 | 0.042252541 |
| Census Region_Northeast | 2015 | 0.767215419 | 0.042840952 |
| Census Region_Northeast | 2016 | 0.686934237 | 0.039129291 |
| Census Region_Northeast | 2017 | 0.752859071 | 0.04154304 |
| Census Region_Northeast | 2018 | 0.935421077 | 0.04601243 |
| Census Region_Northeast | 2019 | 0.980333408 | 0.047677357 |
| Census Region_Northeast | 2020 | 1.186806384 | 0.050722026 |
| Census Region_Northeast | 2021 | 1.366391614 | 0.054049976 |
| Census Region_Northeast | 2022 | 1.334260591 | 0.05249692 |
| Census Region_Northeast | 2023 | 1.298690912 | 0.050595725 |
| Census Region_South | 1999 | 1.117908073 | 0.042280155 |
| Census Region_South | 2000 | 1.081280886 | 0.041587107 |
| Census Region_South | 2001 | 0.994588226 | 0.039256549 |
| Census Region_South | 2002 | 1.075945404 | 0.041076602 |
| Census Region_South | 2003 | 1.073852982 | 0.040462885 |
| Census Region_South | 2004 | 0.997460299 | 0.038246881 |
| Census Region_South | 2005 | 0.944499211 | 0.037018162 |
| Census Region_South | 2006 | 1.024395601 | 0.038237917 |
| Census Region_South | 2007 | 0.896223988 | 0.035118902 |
| Census Region_South | 2008 | 0.952118494 | 0.035947143 |
| Census Region_South | 2009 | 0.947470847 | 0.035668754 |
| Census Region_South | 2010 | 0.938253962 | 0.035616067 |
| Census Region_South | 2011 | 0.929250109 | 0.03492571 |
| Census Region_South | 2012 | 0.900419637 | 0.034140835 |
| Census Region_South | 2013 | 0.875800395 | 0.032425789 |
| Census Region_South | 2014 | 0.848209319 | 0.031409936 |
| Census Region_South | 2015 | 1.042608005 | 0.035214877 |
| Census Region_South | 2016 | 0.942819234 | 0.032300122 |
| Census Region_South | 2017 | 1.08510172 | 0.03470619 |
| Census Region_South | 2018 | 1.050400189 | 0.033426032 |
| Census Region_South | 2019 | 1.161018625 | 0.035259407 |
| Census Region_South | 2020 | 1.416864129 | 0.03761549 |
| Census Region_South | 2021 | 1.814070748 | 0.04362187 |
| Census Region_South | 2022 | 1.673874174 | 0.041151375 |
| Census Region_South | 2023 | 1.661885178 | 0.040633711 |
| Census Region_West | 1999 | 0.866561535 | 0.050086511 |
| Census Region_West | 2000 | 0.955630273 | 0.051486521 |
| Census Region_West | 2001 | 0.842636857 | 0.048504343 |
| Census Region_West | 2002 | 0.898494346 | 0.04957032 |
| Census Region_West | 2003 | 0.903733372 | 0.049332684 |
| Census Region_West | 2004 | 0.937482215 | 0.04957134 |
| Census Region_West | 2005 | 0.794975399 | 0.04498859 |
| Census Region_West | 2006 | 0.881203379 | 0.046535385 |
| Census Region_West | 2007 | 0.818487287 | 0.044251124 |
| Census Region_West | 2008 | 0.915001684 | 0.047080439 |
| Census Region_West | 2009 | 0.907893169 | 0.04590962 |
| Census Region_West | 2010 | 0.898379712 | 0.044622501 |
| Census Region_West | 2011 | 0.939348467 | 0.045971702 |
| Census Region_West | 2012 | 0.835352746 | 0.041658991 |
| Census Region_West | 2013 | 0.840868598 | 0.041479565 |
| Census Region_West | 2014 | 0.881661116 | 0.042210605 |
| Census Region_West | 2015 | 0.980409851 | 0.043327833 |
| Census Region_West | 2016 | 0.992498809 | 0.043522128 |
| Census Region_West | 2017 | 0.987247505 | 0.042254179 |
| Census Region_West | 2018 | 1.10869593 | 0.045140974 |
| Census Region_West | 2019 | 1.125862325 | 0.044991707 |
| Census Region_West | 2020 | 1.385900647 | 0.04889308 |
| Census Region_West | 2021 | 1.827286264 | 0.056595459 |
| Census Region_West | 2022 | 1.688966852 | 0.053279926 |
| Census Region_West | 2023 | 1.55374536 | 0.050883985 |
| Race_Hispanic | 1999 | 0.62721741 | 0.089862542 |
| Race_Hispanic | 2000 | 0.602569706 | 0.084971424 |
| Race_Hispanic | 2001 | 0.586100654 | 0.080125081 |
| Race_Hispanic | 2002 | 0.70859022 | 0.090500554 |
| Race_Hispanic | 2003 | 0.607069263 | 0.078981836 |
| Race_Hispanic | 2004 | 0.509522741 | 0.072386539 |
| Race_Hispanic | 2005 | 0.588804398 | 0.074514403 |
| Race_Hispanic | 2006 | 0.730201797 | 0.079423157 |
| Race_Hispanic | 2007 | 0.48553944 | 0.064144388 |
| Race_Hispanic | 2008 | 0.522019198 | 0.064936842 |
| Race_Hispanic | 2009 | 0.598151955 | 0.068325813 |
| Race_Hispanic | 2010 | 0.705683125 | 0.071517938 |
| Race_Hispanic | 2011 | 0.554085854 | 0.060783023 |
| Race_Hispanic | 2012 | 0.578950147 | 0.059689744 |
| Race_Hispanic | 2013 | 0.494599957 | 0.053348771 |
| Race_Hispanic | 2014 | 0.574320509 | 0.056440128 |
| Race_Hispanic | 2015 | 0.647800819 | 0.057825369 |
| Race_Hispanic | 2016 | 0.5543274 | 0.052549164 |
| Race_Hispanic | 2017 | 0.690616417 | 0.057221333 |
| Race_Hispanic | 2018 | 0.605122797 | 0.05179303 |
| Race_Hispanic | 2019 | 0.726827033 | 0.055957471 |
| Race_Hispanic | 2020 | 0.942258144 | 0.061081231 |
| Race_Hispanic | 2021 | 1.119765963 | 0.066002047 |
| Race_Hispanic | 2022 | 0.976183579 | 0.061064373 |
| Race_Hispanic | 2023 | 0.979271988 | 0.060465663 |
| Race_NH Black | 1999 | 2.347359518 | 0.126555087 |
| Race_NH Black | 2000 | 2.110429984 | 0.118179989 |
| Race_NH Black | 2001 | 2.137529557 | 0.118450085 |
| Race_NH Black | 2002 | 1.988523285 | 0.114197508 |
| Race_NH Black | 2003 | 1.796214711 | 0.106011467 |
| Race_NH Black | 2004 | 1.930700562 | 0.110598105 |
| Race_NH Black | 2005 | 1.951960196 | 0.110374706 |
| Race_NH Black | 2006 | 2.054368767 | 0.112196602 |
| Race_NH Black | 2007 | 1.882581623 | 0.104366482 |
| Race_NH Black | 2008 | 1.930521933 | 0.107405339 |
| Race_NH Black | 2009 | 1.880985049 | 0.103139085 |
| Race_NH Black | 2010 | 1.842066392 | 0.100175769 |
| Race_NH Black | 2011 | 1.586768317 | 0.090825689 |
| Race_NH Black | 2012 | 1.70109106 | 0.093954903 |
| Race_NH Black | 2013 | 1.711807978 | 0.092864577 |
| Race_NH Black | 2014 | 1.583625575 | 0.086314291 |
| Race_NH Black | 2015 | 1.762000003 | 0.090991938 |
| Race_NH Black | 2016 | 1.611807098 | 0.084651181 |
| Race_NH Black | 2017 | 1.886609404 | 0.090073481 |
| Race_NH Black | 2018 | 2.098087625 | 0.095198464 |
| Race_NH Black | 2019 | 2.008340991 | 0.089906 |
| Race_NH Black | 2020 | 2.925148741 | 0.107315061 |
| Race_NH Black | 2021 | 3.760332384 | 0.124120099 |
| Race_NH Black | 2022 | 3.09034617 | 0.109969365 |
| Race_NH Black | 2023 | 3.10841419 | 0.10943102 |
| Race_NH Other | 1999 | 0.417825117 | 0.097286196 |
| Race_NH Other | 2000 | NA | 0.08839355 |
| Race_NH Other | 2001 | 0.479744589 | 0.09817591 |
| Race_NH Other | 2002 | 0.443593376 | 0.097575777 |
| Race_NH Other | 2003 | NA | 0.061220345 |
| Race_NH Other | 2004 | 0.421121672 | 0.083032641 |
| Race_NH Other | 2005 | 0.394923644 | 0.080715063 |
| Race_NH Other | 2006 | 0.390649213 | 0.077091836 |
| Race_NH Other | 2007 | 0.351868809 | 0.070180731 |
| Race_NH Other | 2008 | 0.482081579 | 0.079908007 |
| Race_NH Other | 2009 | 0.421628476 | 0.076685681 |
| Race_NH Other | 2010 | 0.393212751 | 0.070321675 |
| Race_NH Other | 2011 | 0.387090134 | 0.066577373 |
| Race_NH Other | 2012 | 0.37422783 | 0.061496506 |
| Race_NH Other | 2013 | 0.366347993 | 0.059446514 |
| Race_NH Other | 2014 | 0.324694174 | 0.05211034 |
| Race_NH Other | 2015 | 0.336608238 | 0.051863326 |
| Race_NH Other | 2016 | 0.36423913 | 0.056302296 |
| Race_NH Other | 2017 | 0.428691977 | 0.058042869 |
| Race_NH Other | 2018 | 0.48426342 | 0.061411372 |
| Race_NH Other | 2019 | 0.555248838 | 0.063906398 |
| Race_NH Other | 2020 | 0.642837358 | 0.065373058 |
| Race_NH Other | 2021 | 0.765053964 | 0.066328431 |
| Race_NH Other | 2022 | 0.826933626 | 0.067742529 |
| Race_NH Other | 2023 | 0.72270443 | 0.062123295 |
| Race_NH White | 1999 | 0.927110622 | 0.025443087 |
| Race_NH White | 2000 | 0.901985148 | 0.024689903 |
| Race_NH White | 2001 | 0.83817949 | 0.023839413 |
| Race_NH White | 2002 | 0.877528669 | 0.024351749 |
| Race_NH White | 2003 | 0.912981572 | 0.02459382 |
| Race_NH White | 2004 | 0.878165406 | 0.023621367 |
| Race_NH White | 2005 | 0.838697628 | 0.023335162 |
| Race_NH White | 2006 | 0.841457038 | 0.0231735 |
| Race_NH White | 2007 | 0.812695841 | 0.022364203 |
| Race_NH White | 2008 | 0.826445739 | 0.022599768 |
| Race_NH White | 2009 | 0.840007327 | 0.022717542 |
| Race_NH White | 2010 | 0.8428794 | 0.023084157 |
| Race_NH White | 2011 | 0.863827068 | 0.022784115 |
| Race_NH White | 2012 | 0.829434219 | 0.022693426 |
| Race_NH White | 2013 | 0.785889392 | 0.02134298 |
| Race_NH White | 2014 | 0.831912267 | 0.021631655 |
| Race_NH White | 2015 | 0.920846199 | 0.022819474 |
| Race_NH White | 2016 | 0.911756394 | 0.022178043 |
| Race_NH White | 2017 | 0.964737831 | 0.023499291 |
| Race_NH White | 2018 | 1.03389088 | 0.023960198 |
| Race_NH White | 2019 | 1.053337572 | 0.023350058 |
| Race_NH White | 2020 | 1.312599542 | 0.026599258 |
| Race_NH White | 2021 | 1.633006009 | 0.030053487 |
| Race_NH White | 2022 | 1.553895412 | 0.028639042 |
| Race_NH White | 2023 | 1.489303962 | 0.028212478 |
| Sex_Both | 1999 | 0.996837606 | 0.023654883 |
| Sex_Both | 2000 | 0.981092945 | 0.023429731 |
| Sex_Both | 2001 | 0.931155785 | 0.022761402 |
| Sex_Both | 2002 | 0.949914473 | 0.022850584 |
| Sex_Both | 2003 | 0.950718107 | 0.02256782 |
| Sex_Both | 2004 | 0.941125038 | 0.022266174 |
| Sex_Both | 2005 | 0.890399286 | 0.021498274 |
| Sex_Both | 2006 | 0.921204541 | 0.021674446 |
| Sex_Both | 2007 | 0.872259325 | 0.020843565 |
| Sex_Both | 2008 | 0.892700929 | 0.021090249 |
| Sex_Both | 2009 | 0.879095953 | 0.020519442 |
| Sex_Both | 2010 | 0.893322625 | 0.020730222 |
| Sex_Both | 2011 | 0.874918146 | 0.020137008 |
| Sex_Both | 2012 | 0.846041317 | 0.019560864 |
| Sex_Both | 2013 | 0.838209778 | 0.019360563 |
| Sex_Both | 2014 | 0.869272618 | 0.019450766 |
| Sex_Both | 2015 | 0.969937333 | 0.020622408 |
| Sex_Both | 2016 | 0.913894108 | 0.019360762 |
| Sex_Both | 2017 | 1.016227233 | 0.020825881 |
| Sex_Both | 2018 | 1.05317464 | 0.020550905 |
| Sex_Both | 2019 | 1.116675728 | 0.021184912 |
| Sex_Both | 2020 | 1.397956553 | 0.023228454 |
| Sex_Both | 2021 | 1.74367377 | 0.026451782 |
| Sex_Both | 2022 | 1.616069251 | 0.024821112 |
| Sex_Both | 2023 | 1.549437877 | 0.024007313 |
| Sex_Female | 1999 | 0.959004446 | 0.030374855 |
| Sex_Female | 2000 | 0.937207785 | 0.029951915 |
| Sex_Female | 2001 | 0.880208299 | 0.029178045 |
| Sex_Female | 2002 | 0.906926604 | 0.028630341 |
| Sex_Female | 2003 | 0.89120292 | 0.028266818 |
| Sex_Female | 2004 | 0.878332303 | 0.028189913 |
| Sex_Female | 2005 | 0.80893855 | 0.026891318 |
| Sex_Female | 2006 | 0.858120965 | 0.027149368 |
| Sex_Female | 2007 | 0.825566458 | 0.026695067 |
| Sex_Female | 2008 | 0.878329335 | 0.027709958 |
| Sex_Female | 2009 | 0.836726811 | 0.026497692 |
| Sex_Female | 2010 | 0.838864525 | 0.026644604 |
| Sex_Female | 2011 | 0.826515014 | 0.026133949 |
| Sex_Female | 2012 | 0.786217279 | 0.025208108 |
| Sex_Female | 2013 | 0.772397079 | 0.024678651 |
| Sex_Female | 2014 | 0.809969686 | 0.025029305 |
| Sex_Female | 2015 | 0.890638794 | 0.026928327 |
| Sex_Female | 2016 | 0.908142018 | 0.026561581 |
| Sex_Female | 2017 | 0.873402127 | 0.024835396 |
| Sex_Female | 2018 | 1.019519287 | 0.027191131 |
| Sex_Female | 2019 | 1.057062919 | 0.027545107 |
| Sex_Female | 2020 | 1.271865018 | 0.030060032 |
| Sex_Female | 2021 | 1.591703394 | 0.033985694 |
| Sex_Female | 2022 | 1.480299333 | 0.031663782 |
| Sex_Female | 2023 | 1.478731022 | 0.031816299 |
| Sex_Male | 1999 | 1.069226379 | 0.039641328 |
| Sex_Male | 2000 | 1.061931984 | 0.039173981 |
| Sex_Male | 2001 | 1.004160317 | 0.037433928 |
| Sex_Male | 2002 | 0.964401699 | 0.036670719 |
| Sex_Male | 2003 | 1.015029829 | 0.037198208 |
| Sex_Male | 2004 | 1.049224131 | 0.037477496 |
| Sex_Male | 2005 | 1.000542604 | 0.036062547 |
| Sex_Male | 2006 | 0.955360023 | 0.034435043 |
| Sex_Male | 2007 | 0.912848928 | 0.033152283 |
| Sex_Male | 2008 | 0.924218568 | 0.033604629 |
| Sex_Male | 2009 | 0.933195877 | 0.032921308 |
| Sex_Male | 2010 | 0.957731968 | 0.033158326 |
| Sex_Male | 2011 | 0.957404081 | 0.032524157 |
| Sex_Male | 2012 | 0.937401004 | 0.031780346 |
| Sex_Male | 2013 | 0.901624209 | 0.030577829 |
| Sex_Male | 2014 | 0.920956266 | 0.030147952 |
| Sex_Male | 2015 | 1.058850456 | 0.032499725 |
| Sex_Male | 2016 | 0.966359415 | 0.030516011 |
| Sex_Male | 2017 | 1.096284614 | 0.032214067 |
| Sex_Male | 2018 | 1.084431727 | 0.031422537 |
| Sex_Male | 2019 | 1.143571895 | 0.03189409 |
| Sex_Male | 2020 | 1.525449641 | 0.036258017 |
| Sex_Male | 2021 | 1.915643259 | 0.041400477 |
| Sex_Male | 2022 | 1.760128476 | 0.039135368 |
| Sex_Male | 2023 | 1.633540421 | 0.037091854 |
| State_Alabama | 1999 | 1.30449278 | 0.212171725 |
| State_Alabama | 2000 | 1.195187308 | 0.202192752 |
| State_Alabama | 2001 | 1.127933067 | 0.196632217 |
| State_Alabama | 2002 | 0.91519739 | 0.177431107 |
| State_Alabama | 2003 | 1.581873671 | 0.232007522 |
| State_Alabama | 2004 | 1.328578228 | 0.210669564 |
| State_Alabama | 2005 | 1.22885162 | 0.197630892 |
| State_Alabama | 2006 | 0.949764177 | 0.174303407 |
| State_Alabama | 2007 | 0.804175309 | 0.158317923 |
| State_Alabama | 2008 | 1.265276398 | 0.201906884 |
| State_Alabama | 2009 | 0.921622974 | 0.169616143 |
| State_Alabama | 2010 | 0.830565738 | 0.157930153 |
| State_Alabama | 2011 | 1.309528003 | 0.198245145 |
| State_Alabama | 2012 | 0.738605432 | 0.147685365 |
| State_Alabama | 2013 | 0.965780941 | 0.168070587 |
| State_Alabama | 2014 | 0.645454211 | 0.138643208 |
| State_Alabama | 2015 | 1.021893582 | 0.167669095 |
| State_Alabama | 2016 | 1.130723769 | 0.176075476 |
| State_Alabama | 2017 | 0.966561279 | 0.159503893 |
| State_Alabama | 2018 | 0.984671521 | 0.163844175 |
| State_Alabama | 2019 | 0.97921603 | 0.159268603 |
| State_Alabama | 2020 | 1.083208813 | 0.16420758 |
| State_Alabama | 2021 | 1.407424293 | 0.190434721 |
| State_Alabama | 2022 | 1.105937013 | 0.159639954 |
| State_Alabama | 2023 | 1.56521164 | 0.199443236 |
| State_Alaska | 1999 | NA | NA |
| State_Alaska | 2000 | NA | NA |
| State_Alaska | 2001 | NA | NA |
| State_Alaska | 2002 | NA | NA |
| State_Alaska | 2003 | NA | NA |
| State_Alaska | 2004 | NA | NA |
| State_Alaska | 2005 | NA | NA |
| State_Alaska | 2006 | NA | NA |
| State_Alaska | 2007 | NA | NA |
| State_Alaska | 2008 | NA | NA |
| State_Alaska | 2009 | NA | NA |
| State_Alaska | 2010 | NA | NA |
| State_Alaska | 2011 | NA | NA |
| State_Alaska | 2012 | NA | NA |
| State_Alaska | 2013 | NA | NA |
| State_Alaska | 2014 | NA | NA |
| State_Alaska | 2015 | NA | NA |
| State_Alaska | 2016 | NA | NA |
| State_Alaska | 2017 | NA | NA |
| State_Alaska | 2018 | NA | NA |
| State_Alaska | 2019 | NA | NA |
| State_Alaska | 2020 | NA | NA |
| State_Alaska | 2021 | NA | NA |
| State_Alaska | 2022 | NA | NA |
| State_Alaska | 2023 | NA | NA |
| State_Arizona | 1999 | NA | 0.126563509 |
| State_Arizona | 2000 | NA | 0.12194327 |
| State_Arizona | 2001 | NA | 0.112995938 |
| State_Arizona | 2002 | NA | 0.123931911 |
| State_Arizona | 2003 | NA | 0.116733447 |
| State_Arizona | 2004 | 0.605768217 | 0.132517461 |
| State_Arizona | 2005 | NA | 0.099439463 |
| State_Arizona | 2006 | 0.685980244 | 0.132203535 |
| State_Arizona | 2007 | 0.51129037 | 0.111812297 |
| State_Arizona | 2008 | NA | 0.086016432 |
| State_Arizona | 2009 | NA | 0.096705002 |
| State_Arizona | 2010 | 0.596355047 | 0.113448199 |
| State_Arizona | 2011 | 0.906472689 | 0.144049547 |
| State_Arizona | 2012 | 0.617442457 | 0.113314268 |
| State_Arizona | 2013 | 0.691055806 | 0.117748424 |
| State_Arizona | 2014 | 0.654076061 | 0.115312195 |
| State_Arizona | 2015 | 0.823560474 | 0.132268408 |
| State_Arizona | 2016 | 0.841761172 | 0.126998434 |
| State_Arizona | 2017 | 0.664073667 | 0.110078445 |
| State_Arizona | 2018 | 0.478918701 | 0.089704169 |
| State_Arizona | 2019 | 0.607685768 | 0.105201155 |
| State_Arizona | 2020 | 0.99068839 | 0.125543414 |
| State_Arizona | 2021 | 0.973168269 | 0.130507859 |
| State_Arizona | 2022 | 1.181340696 | 0.142937956 |
| State_Arizona | 2023 | 1.116525968 | 0.127953459 |
| State_Arkansas | 1999 | NA | 0.220225649 |
| State_Arkansas | 2000 | 1.08947481 | 0.244156348 |
| State_Arkansas | 2001 | 1.293042662 | 0.265275914 |
| State_Arkansas | 2002 | 1.32552836 | 0.266143469 |
| State_Arkansas | 2003 | 1.339007463 | 0.263095636 |
| State_Arkansas | 2004 | 1.080044578 | 0.237807579 |
| State_Arkansas | 2005 | NA | 0.225631476 |
| State_Arkansas | 2006 | 1.124662837 | 0.236768141 |
| State_Arkansas | 2007 | 1.207188083 | 0.247616259 |
| State_Arkansas | 2008 | 1.018964694 | 0.224152247 |
| State_Arkansas | 2009 | 1.438599842 | 0.264785004 |
| State_Arkansas | 2010 | NA | 0.2099396 |
| State_Arkansas | 2011 | NA | 0.140763644 |
| State_Arkansas | 2012 | 1.017929703 | 0.218821965 |
| State_Arkansas | 2013 | NA | 0.193410513 |
| State_Arkansas | 2014 | NA | 0.203064501 |
| State_Arkansas | 2015 | 1.034361947 | 0.219864268 |
| State_Arkansas | 2016 | 1.220662757 | 0.227561067 |
| State_Arkansas | 2017 | 1.376405287 | 0.243032652 |
| State_Arkansas | 2018 | 1.097721829 | 0.220845197 |
| State_Arkansas | 2019 | 0.984723129 | 0.207253667 |
| State_Arkansas | 2020 | 1.595751691 | 0.257393733 |
| State_Arkansas | 2021 | 1.725798983 | 0.268739991 |
| State_Arkansas | 2022 | 1.627218951 | 0.253956244 |
| State_Arkansas | 2023 | 2.235017537 | 0.306830682 |
| State_California | 1999 | 0.916125479 | 0.070665648 |
| State_California | 2000 | 1.048605571 | 0.074987279 |
| State_California | 2001 | 0.918939747 | 0.069838612 |
| State_California | 2002 | 0.939605055 | 0.069729563 |
| State_California | 2003 | 0.92761942 | 0.069118066 |
| State_California | 2004 | 1.023382307 | 0.071955709 |
| State_California | 2005 | 0.883530347 | 0.065682039 |
| State_California | 2006 | 0.913503138 | 0.066557591 |
| State_California | 2007 | 0.815609278 | 0.062097845 |
| State_California | 2008 | 0.900543433 | 0.064893272 |
| State_California | 2009 | 0.941992041 | 0.065980007 |
| State_California | 2010 | 0.928793537 | 0.064208567 |
| State_California | 2011 | 0.966782585 | 0.064619175 |
| State_California | 2012 | 0.871880173 | 0.06008478 |
| State_California | 2013 | 0.822579122 | 0.058009055 |
| State_California | 2014 | 0.841626903 | 0.05823874 |
| State_California | 2015 | 1.003996322 | 0.063635892 |
| State_California | 2016 | 0.891096599 | 0.058518138 |
| State_California | 2017 | 0.996656665 | 0.060470211 |
| State_California | 2018 | 1.053897094 | 0.062148224 |
| State_California | 2019 | 0.919264041 | 0.05638804 |
| State_California | 2020 | 1.225415859 | 0.06599183 |
| State_California | 2021 | 1.477607432 | 0.072741872 |
| State_California | 2022 | 1.35761629 | 0.067135601 |
| State_California | 2023 | 1.310519889 | 0.066483083 |
| State_Colorado | 1999 | 1.000399096 | 0.219889565 |
| State_Colorado | 2000 | 1.393838545 | 0.255739835 |
| State_Colorado | 2001 | 1.392835301 | 0.255340769 |
| State_Colorado | 2002 | NA | 0.183017262 |
| State_Colorado | 2003 | 1.261704382 | 0.232773678 |
| State_Colorado | 2004 | 0.985899021 | 0.203756694 |
| State_Colorado | 2005 | NA | 0.147136212 |
| State_Colorado | 2006 | 0.973048966 | 0.196491536 |
| State_Colorado | 2007 | 1.215955118 | 0.214326892 |
| State_Colorado | 2008 | 0.96040349 | 0.187598709 |
| State_Colorado | 2009 | 1.510053289 | 0.23405055 |
| State_Colorado | 2010 | 1.181369571 | 0.202304346 |
| State_Colorado | 2011 | 0.965868024 | 0.179298441 |
| State_Colorado | 2012 | 0.964198465 | 0.172923787 |
| State_Colorado | 2013 | 0.768473126 | 0.151228223 |
| State_Colorado | 2014 | 1.010245 | 0.176117392 |
| State_Colorado | 2015 | 1.147046178 | 0.179625902 |
| State_Colorado | 2016 | 1.544495055 | 0.212489138 |
| State_Colorado | 2017 | 1.621293804 | 0.211635532 |
| State_Colorado | 2018 | 1.770401117 | 0.218342086 |
| State_Colorado | 2019 | 2.326826554 | 0.246917716 |
| State_Colorado | 2020 | 2.62177489 | 0.254203584 |
| State_Colorado | 2021 | 3.930738515 | 0.321459049 |
| State_Colorado | 2022 | 3.602063032 | 0.298487883 |
| State_Colorado | 2023 | 2.876521852 | 0.264105706 |
| State_Connecticut | 1999 | NA | 0.169896598 |
| State_Connecticut | 2000 | 0.841991521 | 0.18397209 |
| State_Connecticut | 2001 | 0.811923965 | 0.182024722 |
| State_Connecticut | 2002 | 0.990372705 | 0.199049795 |
| State_Connecticut | 2003 | NA | 0.167147045 |
| State_Connecticut | 2004 | 1.00202415 | 0.197944084 |
| State_Connecticut | 2005 | 1.045660705 | 0.202935286 |
| State_Connecticut | 2006 | 0.755001545 | 0.166087737 |
| State_Connecticut | 2007 | 0.941767694 | 0.186495028 |
| State_Connecticut | 2008 | 0.876861533 | 0.184431115 |
| State_Connecticut | 2009 | 1.058587779 | 0.199866229 |
| State_Connecticut | 2010 | NA | 0.155486417 |
| State_Connecticut | 2011 | 0.750264099 | 0.167500784 |
| State_Connecticut | 2012 | 0.711716364 | 0.162336478 |
| State_Connecticut | 2013 | 0.822033333 | 0.167837755 |
| State_Connecticut | 2014 | 0.696277174 | 0.151676677 |
| State_Connecticut | 2015 | 0.854199035 | 0.176922186 |
| State_Connecticut | 2016 | 0.75716715 | 0.160673787 |
| State_Connecticut | 2017 | 0.78351073 | 0.166624826 |
| State_Connecticut | 2018 | 1.051455837 | 0.186840729 |
| State_Connecticut | 2019 | 0.700731065 | 0.147856035 |
| State_Connecticut | 2020 | 0.92604296 | 0.169538957 |
| State_Connecticut | 2021 | 1.030656564 | 0.184823647 |
| State_Connecticut | 2022 | 1.030706482 | 0.178092934 |
| State_Connecticut | 2023 | 1.219324187 | 0.195919361 |
| State_Delaware | 1999 | NA | NA |
| State_Delaware | 2000 | NA | NA |
| State_Delaware | 2001 | NA | NA |
| State_Delaware | 2002 | NA | NA |
| State_Delaware | 2003 | NA | NA |
| State_Delaware | 2004 | NA | NA |
| State_Delaware | 2005 | NA | NA |
| State_Delaware | 2006 | NA | 0.545713277 |
| State_Delaware | 2007 | NA | NA |
| State_Delaware | 2008 | NA | NA |
| State_Delaware | 2009 | NA | 0.4825008 |
| State_Delaware | 2010 | NA | NA |
| State_Delaware | 2011 | NA | NA |
| State_Delaware | 2012 | NA | NA |
| State_Delaware | 2013 | NA | 0.445921645 |
| State_Delaware | 2014 | NA | NA |
| State_Delaware | 2015 | NA | 0.459911288 |
| State_Delaware | 2016 | NA | NA |
| State_Delaware | 2017 | NA | 0.383582044 |
| State_Delaware | 2018 | NA | NA |
| State_Delaware | 2019 | NA | NA |
| State_Delaware | 2020 | NA | 0.39612746 |
| State_Delaware | 2021 | NA | 0.498144965 |
| State_Delaware | 2022 | NA | 0.467584569 |
| State_Delaware | 2023 | 2.575359343 | 0.583840585 |
| State_District of Columbia | 1999 | NA | 1.112637195 |
| State_District of Columbia | 2000 | NA | NA |
| State_District of Columbia | 2001 | NA | 1.12875685 |
| State_District of Columbia | 2002 | NA | 0.940470753 |
| State_District of Columbia | 2003 | NA | NA |
| State_District of Columbia | 2004 | NA | NA |
| State_District of Columbia | 2005 | NA | NA |
| State_District of Columbia | 2006 | NA | NA |
| State_District of Columbia | 2007 | NA | NA |
| State_District of Columbia | 2008 | NA | NA |
| State_District of Columbia | 2009 | NA | NA |
| State_District of Columbia | 2010 | NA | NA |
| State_District of Columbia | 2011 | NA | NA |
| State_District of Columbia | 2012 | NA | NA |
| State_District of Columbia | 2013 | NA | NA |
| State_District of Columbia | 2014 | NA | NA |
| State_District of Columbia | 2015 | NA | 0.801909646 |
| State_District of Columbia | 2016 | NA | 0.822939022 |
| State_District of Columbia | 2017 | NA | NA |
| State_District of Columbia | 2018 | NA | 0.749920915 |
| State_District of Columbia | 2019 | NA | NA |
| State_District of Columbia | 2020 | NA | 0.808476235 |
| State_District of Columbia | 2021 | NA | 0.959206895 |
| State_District of Columbia | 2022 | NA | 0.84903044 |
| State_District of Columbia | 2023 | 4.548042505 | 0.995710259 |
| State_Florida | 1999 | 0.49207415 | 0.061050681 |
| State_Florida | 2000 | 0.636178075 | 0.069666958 |
| State_Florida | 2001 | 0.659883774 | 0.071079692 |
| State_Florida | 2002 | 0.65950206 | 0.069880138 |
| State_Florida | 2003 | 0.64679297 | 0.068834282 |
| State_Florida | 2004 | 0.548860843 | 0.062072065 |
| State_Florida | 2005 | 0.544066648 | 0.061040324 |
| State_Florida | 2006 | 0.600164673 | 0.064427909 |
| State_Florida | 2007 | 0.481762965 | 0.055449865 |
| State_Florida | 2008 | 0.470273523 | 0.054125125 |
| State_Florida | 2009 | 0.580705137 | 0.062073505 |
| State_Florida | 2010 | 0.66255037 | 0.065396963 |
| State_Florida | 2011 | 0.566776474 | 0.057967243 |
| State_Florida | 2012 | 0.624046756 | 0.061838479 |
| State_Florida | 2013 | 0.535459386 | 0.056902971 |
| State_Florida | 2014 | 0.579105339 | 0.060066433 |
| State_Florida | 2015 | 0.75166224 | 0.06468065 |
| State_Florida | 2016 | 0.65563367 | 0.060319285 |
| State_Florida | 2017 | 0.838050959 | 0.068597033 |
| State_Florida | 2018 | 0.683417452 | 0.060279775 |
| State_Florida | 2019 | 0.831336471 | 0.066392115 |
| State_Florida | 2020 | 1.113906621 | 0.074702233 |
| State_Florida | 2021 | 1.293891783 | 0.081814556 |
| State_Florida | 2022 | 1.195863934 | 0.075128875 |
| State_Florida | 2023 | 1.177312371 | 0.072830518 |
| State_Georgia | 1999 | 1.288905689 | 0.179702467 |
| State_Georgia | 2000 | 1.163514747 | 0.169728387 |
| State_Georgia | 2001 | 0.772280708 | 0.135369997 |
| State_Georgia | 2002 | 1.068263581 | 0.158948408 |
| State_Georgia | 2003 | 1.086042529 | 0.158587547 |
| State_Georgia | 2004 | 0.938426825 | 0.145994776 |
| State_Georgia | 2005 | 0.686413734 | 0.12442446 |
| State_Georgia | 2006 | 1.090418585 | 0.150816436 |
| State_Georgia | 2007 | 0.860685508 | 0.133352117 |
| State_Georgia | 2008 | 0.738160524 | 0.119608771 |
| State_Georgia | 2009 | 0.818225912 | 0.124655107 |
| State_Georgia | 2010 | 0.956398915 | 0.136330241 |
| State_Georgia | 2011 | 0.891498132 | 0.125025412 |
| State_Georgia | 2012 | 0.997890192 | 0.130132209 |
| State_Georgia | 2013 | 0.922067431 | 0.128603732 |
| State_Georgia | 2014 | 0.941945919 | 0.125955213 |
| State_Georgia | 2015 | 0.752312624 | 0.108636477 |
| State_Georgia | 2016 | 0.832309429 | 0.115279582 |
| State_Georgia | 2017 | 0.919462203 | 0.118207803 |
| State_Georgia | 2018 | 0.878588723 | 0.112665796 |
| State_Georgia | 2019 | 0.948988843 | 0.114350354 |
| State_Georgia | 2020 | 0.988380979 | 0.11391636 |
| State_Georgia | 2021 | 1.688263288 | 0.15480003 |
| State_Georgia | 2022 | 1.399371342 | 0.138217974 |
| State_Georgia | 2023 | 1.391722176 | 0.135802695 |
| State_Hawaii | 1999 | NA | NA |
| State_Hawaii | 2000 | NA | NA |
| State_Hawaii | 2001 | NA | NA |
| State_Hawaii | 2002 | NA | NA |
| State_Hawaii | 2003 | NA | NA |
| State_Hawaii | 2004 | NA | NA |
| State_Hawaii | 2005 | NA | NA |
| State_Hawaii | 2006 | NA | NA |
| State_Hawaii | 2007 | NA | NA |
| State_Hawaii | 2008 | NA | NA |
| State_Hawaii | 2009 | NA | NA |
| State_Hawaii | 2010 | NA | NA |
| State_Hawaii | 2011 | NA | NA |
| State_Hawaii | 2012 | NA | NA |
| State_Hawaii | 2013 | NA | NA |
| State_Hawaii | 2014 | NA | NA |
| State_Hawaii | 2015 | NA | NA |
| State_Hawaii | 2016 | NA | NA |
| State_Hawaii | 2017 | NA | NA |
| State_Hawaii | 2018 | NA | NA |
| State_Hawaii | 2019 | NA | NA |
| State_Hawaii | 2020 | NA | 0.244094844 |
| State_Hawaii | 2021 | NA | 0.293494531 |
| State_Hawaii | 2022 | NA | 0.266677813 |
| State_Hawaii | 2023 | NA | 0.299558976 |
| State_Idaho | 1999 | NA | NA |
| State_Idaho | 2000 | NA | NA |
| State_Idaho | 2001 | NA | NA |
| State_Idaho | 2002 | NA | 0.396255209 |
| State_Idaho | 2003 | NA | 0.40015968 |
| State_Idaho | 2004 | NA | NA |
| State_Idaho | 2005 | NA | NA |
| State_Idaho | 2006 | NA | NA |
| State_Idaho | 2007 | NA | NA |
| State_Idaho | 2008 | NA | NA |
| State_Idaho | 2009 | NA | NA |
| State_Idaho | 2010 | NA | 0.353968225 |
| State_Idaho | 2011 | NA | NA |
| State_Idaho | 2012 | NA | NA |
| State_Idaho | 2013 | NA | 0.34001605 |
| State_Idaho | 2014 | NA | NA |
| State_Idaho | 2015 | 1.732742809 | 0.393562824 |
| State_Idaho | 2016 | NA | 0.262907008 |
| State_Idaho | 2017 | NA | 0.268335268 |
| State_Idaho | 2018 | NA | 0.320199094 |
| State_Idaho | 2019 | NA | 0.29755069 |
| State_Idaho | 2020 | NA | 0.27937382 |
| State_Idaho | 2021 | 2.981113963 | 0.480711189 |
| State_Idaho | 2022 | 2.009176425 | 0.372888415 |
| State_Idaho | 2023 | 2.122675196 | 0.38612188 |
| State_Illinois | 1999 | 1.510740125 | 0.141106407 |
| State_Illinois | 2000 | 1.083225125 | 0.11824669 |
| State_Illinois | 2001 | 1.061015657 | 0.115904662 |
| State_Illinois | 2002 | 1.251428474 | 0.126845383 |
| State_Illinois | 2003 | 0.910448431 | 0.108017937 |
| State_Illinois | 2004 | 0.894835663 | 0.108141797 |
| State_Illinois | 2005 | 0.841287174 | 0.103196797 |
| State_Illinois | 2006 | 1.004878581 | 0.110904017 |
| State_Illinois | 2007 | 0.8916382 | 0.105086445 |
| State_Illinois | 2008 | 1.107454358 | 0.116952573 |
| State_Illinois | 2009 | 0.990021957 | 0.110183546 |
| State_Illinois | 2010 | 0.880693152 | 0.102799397 |
| State_Illinois | 2011 | 0.694102884 | 0.089095564 |
| State_Illinois | 2012 | 0.789227802 | 0.095231617 |
| State_Illinois | 2013 | 0.839903658 | 0.097732328 |
| State_Illinois | 2014 | 0.937234476 | 0.101799464 |
| State_Illinois | 2015 | 0.865194032 | 0.099517885 |
| State_Illinois | 2016 | 0.829633374 | 0.094702668 |
| State_Illinois | 2017 | 0.851772363 | 0.095232385 |
| State_Illinois | 2018 | 0.923770481 | 0.098773269 |
| State_Illinois | 2019 | 1.010066368 | 0.103302463 |
| State_Illinois | 2020 | 1.050932223 | 0.106694374 |
| State_Illinois | 2021 | 1.275061623 | 0.11406438 |
| State_Illinois | 2022 | 1.160101097 | 0.10860214 |
| State_Illinois | 2023 | 1.152259252 | 0.106625713 |
| State_Indiana | 1999 | 1.080907023 | 0.168818184 |
| State_Indiana | 2000 | 1.009969421 | 0.16190247 |
| State_Indiana | 2001 | 1.081000778 | 0.166851098 |
| State_Indiana | 2002 | 0.963416063 | 0.156564372 |
| State_Indiana | 2003 | 0.983834111 | 0.15772887 |
| State_Indiana | 2004 | 0.743112872 | 0.136003817 |
| State_Indiana | 2005 | 0.920093128 | 0.149530092 |
| State_Indiana | 2006 | 0.8964826 | 0.145973445 |
| State_Indiana | 2007 | 0.75999551 | 0.131060353 |
| State_Indiana | 2008 | 0.816363453 | 0.136734193 |
| State_Indiana | 2009 | 1.030092302 | 0.155723792 |
| State_Indiana | 2010 | 0.829359767 | 0.137344959 |
| State_Indiana | 2011 | 1.199168747 | 0.164413045 |
| State_Indiana | 2012 | 1.103542033 | 0.157609884 |
| State_Indiana | 2013 | 0.867419742 | 0.137211848 |
| State_Indiana | 2014 | 1.319089237 | 0.168241212 |
| State_Indiana | 2015 | 1.127648902 | 0.150824808 |
| State_Indiana | 2016 | 1.365378342 | 0.167797248 |
| State_Indiana | 2017 | 0.881898761 | 0.132284021 |
| State_Indiana | 2018 | 1.320881995 | 0.162217722 |
| State_Indiana | 2019 | 1.25059137 | 0.153679578 |
| State_Indiana | 2020 | 1.336705887 | 0.164621316 |
| State_Indiana | 2021 | 2.081653901 | 0.20394049 |
| State_Indiana | 2022 | 1.646333625 | 0.176514691 |
| State_Indiana | 2023 | 1.307302315 | 0.15723207 |
| State_Iowa | 1999 | NA | 0.148437875 |
| State_Iowa | 2000 | NA | 0.179138208 |
| State_Iowa | 2001 | NA | 0.175350982 |
| State_Iowa | 2002 | NA | 0.145153587 |
| State_Iowa | 2003 | 1.200707248 | 0.228695541 |
| State_Iowa | 2004 | 0.967477467 | 0.203667305 |
| State_Iowa | 2005 | 0.97120272 | 0.208999454 |
| State_Iowa | 2006 | 0.989353671 | 0.207384347 |
| State_Iowa | 2007 | 0.920273714 | 0.19848117 |
| State_Iowa | 2008 | 0.913062223 | 0.200884808 |
| State_Iowa | 2009 | NA | 0.166791708 |
| State_Iowa | 2010 | 0.823820426 | 0.181750645 |
| State_Iowa | 2011 | 0.998013054 | 0.211912345 |
| State_Iowa | 2012 | 1.095606426 | 0.219567895 |
| State_Iowa | 2013 | 1.068458106 | 0.211015294 |
| State_Iowa | 2014 | 0.995333293 | 0.194346903 |
| State_Iowa | 2015 | 0.800329771 | 0.177636482 |
| State_Iowa | 2016 | 1.292758666 | 0.230502904 |
| State_Iowa | 2017 | 1.30433084 | 0.236965598 |
| State_Iowa | 2018 | 1.047852031 | 0.208089917 |
| State_Iowa | 2019 | 1.502002532 | 0.242552893 |
| State_Iowa | 2020 | 2.253177145 | 0.296466283 |
| State_Iowa | 2021 | 1.799268971 | 0.262361141 |
| State_Iowa | 2022 | 1.619191935 | 0.246959466 |
| State_Iowa | 2023 | 1.439673928 | 0.225780057 |
| State_Kansas | 1999 | NA | 0.176201393 |
| State_Kansas | 2000 | 1.264947011 | 0.264782434 |
| State_Kansas | 2001 | NA | 0.224095355 |
| State_Kansas | 2002 | 1.270467158 | 0.260451253 |
| State_Kansas | 2003 | NA | 0.221488376 |
| State_Kansas | 2004 | NA | NA |
| State_Kansas | 2005 | NA | 0.200078688 |
| State_Kansas | 2006 | NA | 0.167826841 |
| State_Kansas | 2007 | NA | 0.225938827 |
| State_Kansas | 2008 | 0.960327233 | 0.216821684 |
| State_Kansas | 2009 | NA | 0.185836211 |
| State_Kansas | 2010 | NA | 0.168830588 |
| State_Kansas | 2011 | NA | 0.207398859 |
| State_Kansas | 2012 | NA | 0.172929059 |
| State_Kansas | 2013 | NA | 0.211988198 |
| State_Kansas | 2014 | NA | 0.1937572 |
| State_Kansas | 2015 | NA | 0.188688374 |
| State_Kansas | 2016 | 1.160366448 | 0.237018914 |
| State_Kansas | 2017 | 1.193497663 | 0.238240252 |
| State_Kansas | 2018 | NA | 0.206610291 |
| State_Kansas | 2019 | 0.964322094 | 0.20504263 |
| State_Kansas | 2020 | 1.488562769 | 0.257427758 |
| State_Kansas | 2021 | 1.456555319 | 0.2617864 |
| State_Kansas | 2022 | 1.16573253 | 0.220228162 |
| State_Kansas | 2023 | 1.720717547 | 0.269104199 |
| State_Kentucky | 1999 | 1.124682198 | 0.21276984 |
| State_Kentucky | 2000 | 0.984243485 | 0.197133402 |
| State_Kentucky | 2001 | NA | 0.136041861 |
| State_Kentucky | 2002 | 1.070619722 | 0.202935349 |
| State_Kentucky | 2003 | 1.194817892 | 0.214932809 |
| State_Kentucky | 2004 | 1.21595785 | 0.212565823 |
| State_Kentucky | 2005 | 1.137881713 | 0.208826418 |
| State_Kentucky | 2006 | 1.155732753 | 0.206011329 |
| State_Kentucky | 2007 | 0.868495638 | 0.17499706 |
| State_Kentucky | 2008 | 0.838669298 | 0.176192376 |
| State_Kentucky | 2009 | 0.9243798 | 0.175782711 |
| State_Kentucky | 2010 | 0.941791795 | 0.180030386 |
| State_Kentucky | 2011 | 0.916315213 | 0.172709255 |
| State_Kentucky | 2012 | 1.158697334 | 0.199675787 |
| State_Kentucky | 2013 | 0.937385359 | 0.172738018 |
| State_Kentucky | 2014 | 0.986064007 | 0.174647985 |
| State_Kentucky | 2015 | 1.374684819 | 0.210254506 |
| State_Kentucky | 2016 | 1.184655314 | 0.198577613 |
| State_Kentucky | 2017 | 1.268679242 | 0.20466401 |
| State_Kentucky | 2018 | 1.690549625 | 0.222041501 |
| State_Kentucky | 2019 | 1.329093402 | 0.189909117 |
| State_Kentucky | 2020 | 1.798915447 | 0.224121343 |
| State_Kentucky | 2021 | 2.099551469 | 0.244911128 |
| State_Kentucky | 2022 | 1.834087409 | 0.229380787 |
| State_Kentucky | 2023 | 2.212383614 | 0.248663525 |
| State_Louisiana | 1999 | 1.573158751 | 0.246563231 |
| State_Louisiana | 2000 | 1.108563783 | 0.206094318 |
| State_Louisiana | 2001 | 1.048271637 | 0.198519328 |
| State_Louisiana | 2002 | 0.886484919 | 0.181441241 |
| State_Louisiana | 2003 | 0.773450904 | 0.169578817 |
| State_Louisiana | 2004 | 0.803937297 | 0.172827031 |
| State_Louisiana | 2005 | 0.885584367 | 0.177760242 |
| State_Louisiana | 2006 | 1.110869542 | 0.20095251 |
| State_Louisiana | 2007 | 0.776611147 | 0.166845941 |
| State_Louisiana | 2008 | 0.986928891 | 0.188682586 |
| State_Louisiana | 2009 | 0.743311608 | 0.159855203 |
| State_Louisiana | 2010 | NA | 0.156461612 |
| State_Louisiana | 2011 | NA | 0.147534279 |
| State_Louisiana | 2012 | 0.755843187 | 0.159360506 |
| State_Louisiana | 2013 | 0.661057246 | 0.147182784 |
| State_Louisiana | 2014 | NA | 0.136838836 |
| State_Louisiana | 2015 | 0.985964417 | 0.183834815 |
| State_Louisiana | 2016 | 0.77203352 | 0.15425329 |
| State_Louisiana | 2017 | 0.893193141 | 0.168162681 |
| State_Louisiana | 2018 | 0.978750307 | 0.17191771 |
| State_Louisiana | 2019 | 0.9661889 | 0.166541443 |
| State_Louisiana | 2020 | 1.17787052 | 0.184923288 |
| State_Louisiana | 2021 | 1.621130345 | 0.223818258 |
| State_Louisiana | 2022 | 1.195249913 | 0.182778538 |
| State_Louisiana | 2023 | 1.316667868 | 0.196997463 |
| State_Maine | 1999 | NA | 0.394889653 |
| State_Maine | 2000 | NA | 0.352528071 |
| State_Maine | 2001 | NA | 0.353395503 |
| State_Maine | 2002 | NA | NA |
| State_Maine | 2003 | NA | NA |
| State_Maine | 2004 | NA | NA |
| State_Maine | 2005 | NA | 0.297317701 |
| State_Maine | 2006 | NA | NA |
| State_Maine | 2007 | NA | NA |
| State_Maine | 2008 | NA | NA |
| State_Maine | 2009 | NA | NA |
| State_Maine | 2010 | NA | NA |
| State_Maine | 2011 | NA | 0.313521157 |
| State_Maine | 2012 | NA | 0.297805066 |
| State_Maine | 2013 | NA | NA |
| State_Maine | 2014 | NA | NA |
| State_Maine | 2015 | NA | NA |
| State_Maine | 2016 | NA | NA |
| State_Maine | 2017 | NA | NA |
| State_Maine | 2018 | NA | NA |
| State_Maine | 2019 | NA | NA |
| State_Maine | 2020 | NA | NA |
| State_Maine | 2021 | NA | NA |
| State_Maine | 2022 | NA | NA |
| State_Maine | 2023 | NA | NA |
| State_Maryland | 1999 | 1.459423113 | 0.220925154 |
| State_Maryland | 2000 | 1.608477727 | 0.226273315 |
| State_Maryland | 2001 | 1.632157063 | 0.227074908 |
| State_Maryland | 2002 | 1.232245508 | 0.195619183 |
| State_Maryland | 2003 | 1.309536371 | 0.198538307 |
| State_Maryland | 2004 | 1.267844511 | 0.194374071 |
| State_Maryland | 2005 | 1.209003435 | 0.187461057 |
| State_Maryland | 2006 | 1.095484219 | 0.175038214 |
| State_Maryland | 2007 | 0.902869954 | 0.158963932 |
| State_Maryland | 2008 | 1.091935003 | 0.173789121 |
| State_Maryland | 2009 | 1.480476795 | 0.199827329 |
| State_Maryland | 2010 | 1.324011665 | 0.19161171 |
| State_Maryland | 2011 | 1.066835263 | 0.166040797 |
| State_Maryland | 2012 | 1.097723851 | 0.167971475 |
| State_Maryland | 2013 | 1.052283051 | 0.162414309 |
| State_Maryland | 2014 | 0.737405976 | 0.132421134 |
| State_Maryland | 2015 | 1.298903219 | 0.176897627 |
| State_Maryland | 2016 | 1.046651859 | 0.155951579 |
| State_Maryland | 2017 | 1.064226537 | 0.151465851 |
| State_Maryland | 2018 | 1.650749054 | 0.193337441 |
| State_Maryland | 2019 | 1.786609925 | 0.195027517 |
| State_Maryland | 2020 | 3.039560411 | 0.256364662 |
| State_Maryland | 2021 | 3.199381963 | 0.259205846 |
| State_Maryland | 2022 | 2.527682898 | 0.226395078 |
| State_Maryland | 2023 | 2.787914858 | 0.23778968 |
| State_Massachusetts | 1999 | 0.589892338 | 0.115987054 |
| State_Massachusetts | 2000 | 0.724694916 | 0.126348823 |
| State_Massachusetts | 2001 | 0.656623933 | 0.120135184 |
| State_Massachusetts | 2002 | 0.655688764 | 0.120160935 |
| State_Massachusetts | 2003 | 0.727091619 | 0.127081498 |
| State_Massachusetts | 2004 | 0.760602124 | 0.129056003 |
| State_Massachusetts | 2005 | 0.712136725 | 0.125101985 |
| State_Massachusetts | 2006 | 0.730632114 | 0.124296517 |
| State_Massachusetts | 2007 | 1.149774917 | 0.157861903 |
| State_Massachusetts | 2008 | 0.471507642 | 0.099249098 |
| State_Massachusetts | 2009 | 0.598567737 | 0.112069465 |
| State_Massachusetts | 2010 | 0.937779964 | 0.140108881 |
| State_Massachusetts | 2011 | 0.653447387 | 0.115867386 |
| State_Massachusetts | 2012 | 0.613090814 | 0.111475199 |
| State_Massachusetts | 2013 | 0.498626241 | 0.099862219 |
| State_Massachusetts | 2014 | 0.643853482 | 0.111165529 |
| State_Massachusetts | 2015 | 0.641464308 | 0.112466433 |
| State_Massachusetts | 2016 | 0.814231142 | 0.12344746 |
| State_Massachusetts | 2017 | 0.631710731 | 0.103996154 |
| State_Massachusetts | 2018 | 1.023156023 | 0.140322003 |
| State_Massachusetts | 2019 | 0.913388242 | 0.128970863 |
| State_Massachusetts | 2020 | 1.288648214 | 0.147665417 |
| State_Massachusetts | 2021 | 1.44606264 | 0.161566097 |
| State_Massachusetts | 2022 | 1.343667992 | 0.146919683 |
| State_Massachusetts | 2023 | 1.341783133 | 0.150549775 |
| State_Michigan | 1999 | 0.964843193 | 0.125951168 |
| State_Michigan | 2000 | 0.764044705 | 0.110369325 |
| State_Michigan | 2001 | 0.936151307 | 0.122498036 |
| State_Michigan | 2002 | 0.76152333 | 0.110012821 |
| State_Michigan | 2003 | 1.000243256 | 0.122386781 |
| State_Michigan | 2004 | 0.904472928 | 0.118336562 |
| State_Michigan | 2005 | 1.043145947 | 0.125531324 |
| State_Michigan | 2006 | 0.744202183 | 0.103583338 |
| State_Michigan | 2007 | 0.938363989 | 0.118935989 |
| State_Michigan | 2008 | 0.942832372 | 0.115671179 |
| State_Michigan | 2009 | 0.824164839 | 0.110424653 |
| State_Michigan | 2010 | 1.011050973 | 0.119558159 |
| State_Michigan | 2011 | 0.871153892 | 0.110194804 |
| State_Michigan | 2012 | 0.797937759 | 0.106300804 |
| State_Michigan | 2013 | 0.776804984 | 0.103514799 |
| State_Michigan | 2014 | 0.74010712 | 0.099120404 |
| State_Michigan | 2015 | 0.853143121 | 0.105022051 |
| State_Michigan | 2016 | 0.743247399 | 0.098902774 |
| State_Michigan | 2017 | 1.045953136 | 0.118903501 |
| State_Michigan | 2018 | 1.043161114 | 0.115141589 |
| State_Michigan | 2019 | 1.037552711 | 0.115278903 |
| State_Michigan | 2020 | 1.335184975 | 0.126684394 |
| State_Michigan | 2021 | 1.735109931 | 0.148232641 |
| State_Michigan | 2022 | 1.86282757 | 0.150850524 |
| State_Michigan | 2023 | 1.480530119 | 0.133196042 |
| State_Minnesota | 1999 | 0.689936727 | 0.151457139 |
| State_Minnesota | 2000 | 0.688844855 | 0.147843411 |
| State_Minnesota | 2001 | 1.270622774 | 0.199671373 |
| State_Minnesota | 2002 | 0.815950048 | 0.157908583 |
| State_Minnesota | 2003 | 0.701828925 | 0.147228039 |
| State_Minnesota | 2004 | 0.840405516 | 0.157032801 |
| State_Minnesota | 2005 | 0.834029439 | 0.158978096 |
| State_Minnesota | 2006 | 1.047691775 | 0.178215444 |
| State_Minnesota | 2007 | 0.843392223 | 0.155481104 |
| State_Minnesota | 2008 | 0.694775641 | 0.138124738 |
| State_Minnesota | 2009 | 0.896827537 | 0.157643254 |
| State_Minnesota | 2010 | 0.7332709 | 0.137988826 |
| State_Minnesota | 2011 | 0.887130846 | 0.15265426 |
| State_Minnesota | 2012 | 0.89369979 | 0.148888541 |
| State_Minnesota | 2013 | 0.947043058 | 0.154308181 |
| State_Minnesota | 2014 | 0.979832347 | 0.157039925 |
| State_Minnesota | 2015 | 1.15013476 | 0.16511656 |
| State_Minnesota | 2016 | 1.444614622 | 0.186558522 |
| State_Minnesota | 2017 | 1.456715078 | 0.188023028 |
| State_Minnesota | 2018 | 1.87336808 | 0.207304846 |
| State_Minnesota | 2019 | 1.575995887 | 0.186190943 |
| State_Minnesota | 2020 | 2.34140985 | 0.22708257 |
| State_Minnesota | 2021 | 2.674368989 | 0.245694617 |
| State_Minnesota | 2022 | 2.743458078 | 0.244573461 |
| State_Minnesota | 2023 | 2.660233653 | 0.238938003 |
| State_Mississippi | 1999 | 1.595101678 | 0.301766029 |
| State_Mississippi | 2000 | 1.320897049 | 0.275690351 |
| State_Mississippi | 2001 | NA | 0.231990015 |
| State_Mississippi | 2002 | 1.17855681 | 0.257653177 |
| State_Mississippi | 2003 | 1.396450919 | 0.279798458 |
| State_Mississippi | 2004 | 1.469927269 | 0.283802156 |
| State_Mississippi | 2005 | 1.709784278 | 0.304135391 |
| State_Mississippi | 2006 | 1.30429334 | 0.267703066 |
| State_Mississippi | 2007 | 1.629937211 | 0.299044751 |
| State_Mississippi | 2008 | 1.473970896 | 0.28132079 |
| State_Mississippi | 2009 | 1.349394527 | 0.270696694 |
| State_Mississippi | 2010 | 1.147832937 | 0.245908207 |
| State_Mississippi | 2011 | 1.10418725 | 0.238012167 |
| State_Mississippi | 2012 | 1.129171317 | 0.232241342 |
| State_Mississippi | 2013 | 1.501130503 | 0.276340084 |
| State_Mississippi | 2014 | 1.320124853 | 0.254019962 |
| State_Mississippi | 2015 | 1.690725868 | 0.281258336 |
| State_Mississippi | 2016 | 0.963796898 | 0.213446648 |
| State_Mississippi | 2017 | 1.596165191 | 0.273076587 |
| State_Mississippi | 2018 | 1.289789662 | 0.248726456 |
| State_Mississippi | 2019 | 1.748623942 | 0.279037195 |
| State_Mississippi | 2020 | 1.535004422 | 0.256878047 |
| State_Mississippi | 2021 | 2.358457994 | 0.319261684 |
| State_Mississippi | 2022 | 2.387136425 | 0.327066355 |
| State_Mississippi | 2023 | 2.434465048 | 0.328824353 |
| State_Missouri | 1999 | 1.292673209 | 0.183001441 |
| State_Missouri | 2000 | 0.918066255 | 0.155434484 |
| State_Missouri | 2001 | 1.118926103 | 0.171119603 |
| State_Missouri | 2002 | 0.961627042 | 0.15614838 |
| State_Missouri | 2003 | 1.112775726 | 0.16612182 |
| State_Missouri | 2004 | 1.162123402 | 0.167975329 |
| State_Missouri | 2005 | 0.960933501 | 0.154208449 |
| State_Missouri | 2006 | 1.035964893 | 0.161098818 |
| State_Missouri | 2007 | 0.93930259 | 0.151927642 |
| State_Missouri | 2008 | 0.906728281 | 0.145712623 |
| State_Missouri | 2009 | 1.021721756 | 0.15571875 |
| State_Missouri | 2010 | 1.00212594 | 0.148446286 |
| State_Missouri | 2011 | 0.924872121 | 0.145519246 |
| State_Missouri | 2012 | 0.787826843 | 0.132392999 |
| State_Missouri | 2013 | 0.795484432 | 0.130177005 |
| State_Missouri | 2014 | 0.817763537 | 0.139253316 |
| State_Missouri | 2015 | 0.705430851 | 0.128329247 |
| State_Missouri | 2016 | 1.161152409 | 0.158817309 |
| State_Missouri | 2017 | 0.965608074 | 0.138061697 |
| State_Missouri | 2018 | 0.886337776 | 0.133840285 |
| State_Missouri | 2019 | 0.824931346 | 0.128912318 |
| State_Missouri | 2020 | 1.319373775 | 0.164049581 |
| State_Missouri | 2021 | 1.427893254 | 0.166995117 |
| State_Missouri | 2022 | 1.427441699 | 0.166187702 |
| State_Missouri | 2023 | 1.609926183 | 0.181561658 |
| State_Montana | 1999 | NA | NA |
| State_Montana | 2000 | NA | NA |
| State_Montana | 2001 | NA | 0.492141783 |
| State_Montana | 2002 | NA | NA |
| State_Montana | 2003 | NA | NA |
| State_Montana | 2004 | NA | NA |
| State_Montana | 2005 | NA | NA |
| State_Montana | 2006 | NA | NA |
| State_Montana | 2007 | NA | NA |
| State_Montana | 2008 | NA | NA |
| State_Montana | 2009 | NA | NA |
| State_Montana | 2010 | NA | NA |
| State_Montana | 2011 | NA | NA |
| State_Montana | 2012 | NA | NA |
| State_Montana | 2013 | NA | 0.422547624 |
| State_Montana | 2014 | NA | NA |
| State_Montana | 2015 | NA | 0.411063233 |
| State_Montana | 2016 | NA | 0.355718559 |
| State_Montana | 2017 | NA | 0.346878514 |
| State_Montana | 2018 | NA | 0.339861615 |
| State_Montana | 2019 | NA | 0.386908579 |
| State_Montana | 2020 | NA | 0.413408083 |
| State_Montana | 2021 | NA | 0.441448232 |
| State_Montana | 2022 | NA | 0.422203128 |
| State_Montana | 2023 | NA | 0.417094783 |
| State_Nebraska | 1999 | NA | 0.344688411 |
| State_Nebraska | 2000 | NA | NA |
| State_Nebraska | 2001 | NA | NA |
| State_Nebraska | 2002 | NA | 0.311843551 |
| State_Nebraska | 2003 | NA | 0.286636103 |
| State_Nebraska | 2004 | NA | 0.299732371 |
| State_Nebraska | 2005 | NA | 0.325366565 |
| State_Nebraska | 2006 | NA | 0.291060633 |
| State_Nebraska | 2007 | NA | 0.302658237 |
| State_Nebraska | 2008 | NA | 0.261893098 |
| State_Nebraska | 2009 | NA | 0.260133662 |
| State_Nebraska | 2010 | NA | NA |
| State_Nebraska | 2011 | NA | 0.254598699 |
| State_Nebraska | 2012 | NA | 0.332201547 |
| State_Nebraska | 2013 | 1.401961011 | 0.317546261 |
| State_Nebraska | 2014 | NA | 0.294484597 |
| State_Nebraska | 2015 | NA | 0.292022752 |
| State_Nebraska | 2016 | NA | 0.309525111 |
| State_Nebraska | 2017 | NA | 0.301587121 |
| State_Nebraska | 2018 | 1.929143257 | 0.364248389 |
| State_Nebraska | 2019 | 1.740810482 | 0.348510419 |
| State_Nebraska | 2020 | 2.023609158 | 0.37869766 |
| State_Nebraska | 2021 | 2.430236488 | 0.426237164 |
| State_Nebraska | 2022 | 2.31964389 | 0.38633769 |
| State_Nebraska | 2023 | 1.754559911 | 0.351412764 |
| State_Nevada | 1999 | NA | NA |
| State_Nevada | 2000 | NA | 0.292168377 |
| State_Nevada | 2001 | NA | NA |
| State_Nevada | 2002 | NA | NA |
| State_Nevada | 2003 | NA | NA |
| State_Nevada | 2004 | NA | 0.274489425 |
| State_Nevada | 2005 | NA | NA |
| State_Nevada | 2006 | NA | 0.232724167 |
| State_Nevada | 2007 | NA | 0.250281095 |
| State_Nevada | 2008 | NA | 0.272542294 |
| State_Nevada | 2009 | NA | NA |
| State_Nevada | 2010 | NA | 0.241998204 |
| State_Nevada | 2011 | NA | 0.190269966 |
| State_Nevada | 2012 | NA | 0.229407641 |
| State_Nevada | 2013 | NA | NA |
| State_Nevada | 2014 | NA | 0.191338559 |
| State_Nevada | 2015 | NA | NA |
| State_Nevada | 2016 | NA | 0.148826822 |
| State_Nevada | 2017 | NA | 0.187584 |
| State_Nevada | 2018 | NA | 0.181783267 |
| State_Nevada | 2019 | NA | 0.19174835 |
| State_Nevada | 2020 | 1.088768355 | 0.215635733 |
| State_Nevada | 2021 | 1.557005097 | 0.25789027 |
| State_Nevada | 2022 | 1.66426483 | 0.270610372 |
| State_Nevada | 2023 | 1.084614023 | 0.206220376 |
| State_New Hampshire | 1999 | NA | NA |
| State_New Hampshire | 2000 | NA | NA |
| State_New Hampshire | 2001 | NA | 0.402083579 |
| State_New Hampshire | 2002 | NA | NA |
| State_New Hampshire | 2003 | NA | 0.436468678 |
| State_New Hampshire | 2004 | NA | NA |
| State_New Hampshire | 2005 | NA | NA |
| State_New Hampshire | 2006 | NA | 0.384324794 |
| State_New Hampshire | 2007 | NA | NA |
| State_New Hampshire | 2008 | NA | 0.383358733 |
| State_New Hampshire | 2009 | NA | NA |
| State_New Hampshire | 2010 | NA | NA |
| State_New Hampshire | 2011 | NA | 0.332497858 |
| State_New Hampshire | 2012 | NA | NA |
| State_New Hampshire | 2013 | NA | NA |
| State_New Hampshire | 2014 | NA | 0.279955123 |
| State_New Hampshire | 2015 | NA | NA |
| State_New Hampshire | 2016 | NA | NA |
| State_New Hampshire | 2017 | NA | 0.329643764 |
| State_New Hampshire | 2018 | NA | 0.310259825 |
| State_New Hampshire | 2019 | NA | 0.335340482 |
| State_New Hampshire | 2020 | 1.767076888 | 0.418945997 |
| State_New Hampshire | 2021 | 1.628444517 | 0.366029409 |
| State_New Hampshire | 2022 | NA | 0.325651734 |
| State_New Hampshire | 2023 | 1.676118851 | 0.365540767 |
| State_New Jersey | 1999 | 0.780255653 | 0.117677549 |
| State_New Jersey | 2000 | 0.728245124 | 0.112698274 |
| State_New Jersey | 2001 | 0.70155748 | 0.109814825 |
| State_New Jersey | 2002 | 0.569618629 | 0.101115552 |
| State_New Jersey | 2003 | 0.694269189 | 0.108570564 |
| State_New Jersey | 2004 | 0.624630497 | 0.104716953 |
| State_New Jersey | 2005 | 0.932713335 | 0.123829404 |
| State_New Jersey | 2006 | 0.697339421 | 0.10559847 |
| State_New Jersey | 2007 | 0.582515955 | 0.099280902 |
| State_New Jersey | 2008 | 0.475111698 | 0.087484273 |
| State_New Jersey | 2009 | 0.531719532 | 0.092088064 |
| State_New Jersey | 2010 | 0.804069798 | 0.112436745 |
| State_New Jersey | 2011 | 0.761325008 | 0.11114171 |
| State_New Jersey | 2012 | 0.99375555 | 0.125801187 |
| State_New Jersey | 2013 | 0.733472382 | 0.106680882 |
| State_New Jersey | 2014 | 0.712103238 | 0.102028399 |
| State_New Jersey | 2015 | 0.754631208 | 0.104326888 |
| State_New Jersey | 2016 | 0.693070012 | 0.099838302 |
| State_New Jersey | 2017 | 0.817710869 | 0.110904473 |
| State_New Jersey | 2018 | 0.953413777 | 0.118485745 |
| State_New Jersey | 2019 | 0.914168907 | 0.114707024 |
| State_New Jersey | 2020 | 0.772162849 | 0.101482745 |
| State_New Jersey | 2021 | 1.170835016 | 0.125957776 |
| State_New Jersey | 2022 | 1.220632405 | 0.128419437 |
| State_New Jersey | 2023 | 1.332516278 | 0.131105145 |
| State_New Mexico | 1999 | NA | NA |
| State_New Mexico | 2000 | NA | NA |
| State_New Mexico | 2001 | NA | NA |
| State_New Mexico | 2002 | NA | NA |
| State_New Mexico | 2003 | NA | NA |
| State_New Mexico | 2004 | NA | NA |
| State_New Mexico | 2005 | NA | NA |
| State_New Mexico | 2006 | NA | 0.310665662 |
| State_New Mexico | 2007 | NA | 0.244126696 |
| State_New Mexico | 2008 | NA | 0.251283807 |
| State_New Mexico | 2009 | 1.460655173 | 0.329640506 |
| State_New Mexico | 2010 | NA | 0.292897048 |
| State_New Mexico | 2011 | NA | 0.212241167 |
| State_New Mexico | 2012 | NA | NA |
| State_New Mexico | 2013 | NA | NA |
| State_New Mexico | 2014 | NA | 0.240157626 |
| State_New Mexico | 2015 | NA | NA |
| State_New Mexico | 2016 | NA | NA |
| State_New Mexico | 2017 | NA | 0.221899779 |
| State_New Mexico | 2018 | NA | 0.248596664 |
| State_New Mexico | 2019 | NA | 0.217191708 |
| State_New Mexico | 2020 | 1.189856391 | 0.276110548 |
| State_New Mexico | 2021 | 1.652934735 | 0.319511203 |
| State_New Mexico | 2022 | 1.18130083 | 0.255291616 |
| State_New Mexico | 2023 | 1.361822774 | 0.277451984 |
| State_New York | 1999 | 0.664682316 | 0.074160757 |
| State_New York | 2000 | 0.699500137 | 0.074589849 |
| State_New York | 2001 | 0.556888599 | 0.065666283 |
| State_New York | 2002 | 0.643901943 | 0.071426534 |
| State_New York | 2003 | 0.704137057 | 0.074434649 |
| State_New York | 2004 | 0.606953668 | 0.068369156 |
| State_New York | 2005 | 0.567901431 | 0.065866436 |
| State_New York | 2006 | 0.590234444 | 0.066615475 |
| State_New York | 2007 | 0.636004639 | 0.070129122 |
| State_New York | 2008 | 0.619229872 | 0.067446924 |
| State_New York | 2009 | 0.623074447 | 0.066800371 |
| State_New York | 2010 | 0.539719605 | 0.062490706 |
| State_New York | 2011 | 0.655757927 | 0.067966273 |
| State_New York | 2012 | 0.634295405 | 0.066990508 |
| State_New York | 2013 | 0.5614519 | 0.060871525 |
| State_New York | 2014 | 0.641217781 | 0.065316705 |
| State_New York | 2015 | 0.589077356 | 0.064494159 |
| State_New York | 2016 | 0.47896993 | 0.055807868 |
| State_New York | 2017 | 0.602635183 | 0.063058189 |
| State_New York | 2018 | 0.836738649 | 0.075669381 |
| State_New York | 2019 | 0.898712877 | 0.076148785 |
| State_New York | 2020 | 1.086354385 | 0.08226249 |
| State_New York | 2021 | 1.177752263 | 0.085900201 |
| State_New York | 2022 | 1.097300856 | 0.082367036 |
| State_New York | 2023 | 1.119276038 | 0.080236806 |
| State_North Carolina | 1999 | 1.74755903 | 0.190300749 |
| State_North Carolina | 2000 | 1.04318355 | 0.14656611 |
| State_North Carolina | 2001 | 1.28899202 | 0.16302313 |
| State_North Carolina | 2002 | 1.725116799 | 0.185598438 |
| State_North Carolina | 2003 | 1.471038168 | 0.169431626 |
| State_North Carolina | 2004 | 1.331636608 | 0.157875461 |
| State_North Carolina | 2005 | 1.442750635 | 0.165228222 |
| State_North Carolina | 2006 | 1.34911189 | 0.155941198 |
| State_North Carolina | 2007 | 0.999268447 | 0.131011856 |
| State_North Carolina | 2008 | 1.359329456 | 0.152704065 |
| State_North Carolina | 2009 | 1.414227566 | 0.154450201 |
| State_North Carolina | 2010 | 1.049734957 | 0.129244959 |
| State_North Carolina | 2011 | 0.96314664 | 0.121643433 |
| State_North Carolina | 2012 | 1.081557701 | 0.130343238 |
| State_North Carolina | 2013 | 1.116716095 | 0.126622132 |
| State_North Carolina | 2014 | 1.034767361 | 0.121201633 |
| State_North Carolina | 2015 | 1.149639675 | 0.12706406 |
| State_North Carolina | 2016 | 1.029344057 | 0.116145888 |
| State_North Carolina | 2017 | 1.206987887 | 0.127343549 |
| State_North Carolina | 2018 | 0.825514202 | 0.104069177 |
| State_North Carolina | 2019 | 1.072386368 | 0.1178603 |
| State_North Carolina | 2020 | 1.325980877 | 0.131355401 |
| State_North Carolina | 2021 | 2.009596214 | 0.160346544 |
| State_North Carolina | 2022 | 1.890027571 | 0.149455507 |
| State_North Carolina | 2023 | 1.840360635 | 0.145906454 |
| State_North Dakota | 1999 | NA | NA |
| State_North Dakota | 2000 | NA | NA |
| State_North Dakota | 2001 | NA | NA |
| State_North Dakota | 2002 | NA | NA |
| State_North Dakota | 2003 | NA | NA |
| State_North Dakota | 2004 | NA | NA |
| State_North Dakota | 2005 | NA | NA |
| State_North Dakota | 2006 | NA | NA |
| State_North Dakota | 2007 | NA | NA |
| State_North Dakota | 2008 | NA | NA |
| State_North Dakota | 2009 | NA | NA |
| State_North Dakota | 2010 | NA | NA |
| State_North Dakota | 2011 | NA | NA |
| State_North Dakota | 2012 | NA | NA |
| State_North Dakota | 2013 | NA | NA |
| State_North Dakota | 2014 | NA | NA |
| State_North Dakota | 2015 | NA | NA |
| State_North Dakota | 2016 | NA | NA |
| State_North Dakota | 2017 | NA | NA |
| State_North Dakota | 2018 | NA | NA |
| State_North Dakota | 2019 | NA | NA |
| State_North Dakota | 2020 | NA | 0.618428364 |
| State_North Dakota | 2021 | NA | NA |
| State_North Dakota | 2022 | NA | NA |
| State_North Dakota | 2023 | NA | NA |
| State_Ohio | 1999 | 1.360723198 | 0.133539982 |
| State_Ohio | 2000 | 1.170381476 | 0.125037006 |
| State_Ohio | 2001 | 1.067843998 | 0.118248921 |
| State_Ohio | 2002 | 1.223250576 | 0.127089057 |
| State_Ohio | 2003 | 1.27034116 | 0.128030532 |
| State_Ohio | 2004 | 1.412126344 | 0.132974013 |
| State_Ohio | 2005 | 1.099601774 | 0.117600578 |
| State_Ohio | 2006 | 1.410283109 | 0.131298207 |
| State_Ohio | 2007 | 1.167991781 | 0.118296884 |
| State_Ohio | 2008 | 1.152082533 | 0.117372627 |
| State_Ohio | 2009 | 1.179531137 | 0.120580116 |
| State_Ohio | 2010 | 1.163843056 | 0.119225983 |
| State_Ohio | 2011 | 1.137222527 | 0.115117661 |
| State_Ohio | 2012 | 0.86522629 | 0.099409135 |
| State_Ohio | 2013 | 0.91063515 | 0.102116201 |
| State_Ohio | 2014 | 0.926922144 | 0.102654575 |
| State_Ohio | 2015 | 1.003268708 | 0.105965274 |
| State_Ohio | 2016 | 1.031677458 | 0.106836469 |
| State_Ohio | 2017 | 1.020822329 | 0.106296732 |
| State_Ohio | 2018 | 1.025906321 | 0.102468933 |
| State_Ohio | 2019 | 0.981309972 | 0.100239599 |
| State_Ohio | 2020 | 1.488823209 | 0.127890601 |
| State_Ohio | 2021 | 2.042505062 | 0.147577903 |
| State_Ohio | 2022 | 1.5417456 | 0.12591678 |
| State_Ohio | 2023 | 1.489745019 | 0.125252976 |
| State_Oklahoma | 1999 | NA | 0.161366693 |
| State_Oklahoma | 2000 | 1.034564174 | 0.211385619 |
| State_Oklahoma | 2001 | 0.945762546 | 0.201874702 |
| State_Oklahoma | 2002 | 1.285184771 | 0.235291575 |
| State_Oklahoma | 2003 | 1.136466513 | 0.218990416 |
| State_Oklahoma | 2004 | 0.957727096 | 0.200128483 |
| State_Oklahoma | 2005 | NA | 0.17825242 |
| State_Oklahoma | 2006 | 0.805433594 | 0.180713091 |
| State_Oklahoma | 2007 | NA | 0.178584752 |
| State_Oklahoma | 2008 | 0.78971068 | 0.177387565 |
| State_Oklahoma | 2009 | NA | NA |
| State_Oklahoma | 2010 | NA | 0.143817267 |
| State_Oklahoma | 2011 | 1.03848486 | 0.197894306 |
| State_Oklahoma | 2012 | 0.734672248 | 0.165513331 |
| State_Oklahoma | 2013 | 0.816665162 | 0.175968953 |
| State_Oklahoma | 2014 | 0.906080184 | 0.179811495 |
| State_Oklahoma | 2015 | 1.327090974 | 0.214723442 |
| State_Oklahoma | 2016 | 1.35202077 | 0.216127897 |
| State_Oklahoma | 2017 | 1.254546018 | 0.209498005 |
| State_Oklahoma | 2018 | 1.029880813 | 0.184011243 |
| State_Oklahoma | 2019 | 1.446431322 | 0.223920433 |
| State_Oklahoma | 2020 | 1.65956275 | 0.242692034 |
| State_Oklahoma | 2021 | 2.851068623 | 0.316597696 |
| State_Oklahoma | 2022 | 1.937936677 | 0.253953009 |
| State_Oklahoma | 2023 | 1.207238576 | 0.198666349 |
| State_Oregon | 1999 | NA | 0.192227025 |
| State_Oregon | 2000 | NA | 0.191546084 |
| State_Oregon | 2001 | NA | 0.180889543 |
| State_Oregon | 2002 | 1.122502503 | 0.216278706 |
| State_Oregon | 2003 | 0.970903319 | 0.198447479 |
| State_Oregon | 2004 | 0.849097053 | 0.186186021 |
| State_Oregon | 2005 | 0.952861238 | 0.195292119 |
| State_Oregon | 2006 | 1.014696229 | 0.196423713 |
| State_Oregon | 2007 | NA | 0.166785408 |
| State_Oregon | 2008 | 1.5991188 | 0.246240395 |
| State_Oregon | 2009 | 0.823937173 | 0.173655049 |
| State_Oregon | 2010 | NA | 0.159830103 |
| State_Oregon | 2011 | 0.974168913 | 0.184882662 |
| State_Oregon | 2012 | 0.889151833 | 0.173744741 |
| State_Oregon | 2013 | 0.861097603 | 0.172951677 |
| State_Oregon | 2014 | 1.00806914 | 0.178835749 |
| State_Oregon | 2015 | 1.12236445 | 0.188022742 |
| State_Oregon | 2016 | 1.017646121 | 0.177228592 |
| State_Oregon | 2017 | 1.101980259 | 0.187756079 |
| State_Oregon | 2018 | 1.343905148 | 0.201219842 |
| State_Oregon | 2019 | 1.534394504 | 0.212104843 |
| State_Oregon | 2020 | 1.391523082 | 0.200557725 |
| State_Oregon | 2021 | 2.412088306 | 0.27014029 |
| State_Oregon | 2022 | 2.290986017 | 0.25425286 |
| State_Oregon | 2023 | 2.809687735 | 0.287852475 |
| State_Pennsylvania | 1999 | 1.090864702 | 0.106819556 |
| State_Pennsylvania | 2000 | 1.130651019 | 0.111114579 |
| State_Pennsylvania | 2001 | 0.928773365 | 0.102004678 |
| State_Pennsylvania | 2002 | 0.880576757 | 0.096260693 |
| State_Pennsylvania | 2003 | 0.844755564 | 0.0935716 |
| State_Pennsylvania | 2004 | 1.06516177 | 0.104410795 |
| State_Pennsylvania | 2005 | 1.009871984 | 0.102087887 |
| State_Pennsylvania | 2006 | 0.694362272 | 0.083452515 |
| State_Pennsylvania | 2007 | 0.980102972 | 0.097058823 |
| State_Pennsylvania | 2008 | 0.85089188 | 0.093264611 |
| State_Pennsylvania | 2009 | 0.8803857 | 0.09423161 |
| State_Pennsylvania | 2010 | 0.957100822 | 0.097294776 |
| State_Pennsylvania | 2011 | 0.855078829 | 0.093146048 |
| State_Pennsylvania | 2012 | 1.013403948 | 0.098852192 |
| State_Pennsylvania | 2013 | 0.738323049 | 0.082422035 |
| State_Pennsylvania | 2014 | 1.005046531 | 0.099305304 |
| State_Pennsylvania | 2015 | 1.045015713 | 0.101581672 |
| State_Pennsylvania | 2016 | 0.883039361 | 0.091763561 |
| State_Pennsylvania | 2017 | 0.915511713 | 0.093047595 |
| State_Pennsylvania | 2018 | 1.091267235 | 0.098967125 |
| State_Pennsylvania | 2019 | 1.147129003 | 0.103632814 |
| State_Pennsylvania | 2020 | 1.530239172 | 0.119526387 |
| State_Pennsylvania | 2021 | 1.853839687 | 0.127720169 |
| State_Pennsylvania | 2022 | 1.868262526 | 0.130859927 |
| State_Pennsylvania | 2023 | 1.57751821 | 0.11530954 |
| State_Rhode Island | 1999 | NA | NA |
| State_Rhode Island | 2000 | NA | NA |
| State_Rhode Island | 2001 | NA | NA |
| State_Rhode Island | 2002 | NA | NA |
| State_Rhode Island | 2003 | NA | 0.435762752 |
| State_Rhode Island | 2004 | NA | NA |
| State_Rhode Island | 2005 | NA | NA |
| State_Rhode Island | 2006 | NA | NA |
| State_Rhode Island | 2007 | NA | 0.391617885 |
| State_Rhode Island | 2008 | NA | NA |
| State_Rhode Island | 2009 | NA | 0.398059438 |
| State_Rhode Island | 2010 | NA | 0.416862204 |
| State_Rhode Island | 2011 | NA | NA |
| State_Rhode Island | 2012 | NA | 0.383567172 |
| State_Rhode Island | 2013 | NA | 0.455823168 |
| State_Rhode Island | 2014 | NA | NA |
| State_Rhode Island | 2015 | NA | 0.376070672 |
| State_Rhode Island | 2016 | NA | 0.366448981 |
| State_Rhode Island | 2017 | NA | NA |
| State_Rhode Island | 2018 | NA | NA |
| State_Rhode Island | 2019 | NA | NA |
| State_Rhode Island | 2020 | NA | 0.369527181 |
| State_Rhode Island | 2021 | NA | 0.376218353 |
| State_Rhode Island | 2022 | 2.176593354 | 0.484438726 |
| State_Rhode Island | 2023 | NA | 0.395096608 |
| State_South Carolina | 1999 | 1.690195453 | 0.268602577 |
| State_South Carolina | 2000 | 1.689748107 | 0.268141169 |
| State_South Carolina | 2001 | 1.268147453 | 0.225161967 |
| State_South Carolina | 2002 | 1.33934924 | 0.230875382 |
| State_South Carolina | 2003 | 1.233700924 | 0.2163664 |
| State_South Carolina | 2004 | 1.309691906 | 0.225890697 |
| State_South Carolina | 2005 | 1.229244935 | 0.212953391 |
| State_South Carolina | 2006 | 1.211410905 | 0.21233769 |
| State_South Carolina | 2007 | 1.704011857 | 0.243629824 |
| State_South Carolina | 2008 | 1.320017159 | 0.21063807 |
| State_South Carolina | 2009 | 1.188287352 | 0.196889697 |
| State_South Carolina | 2010 | 1.195813498 | 0.195642568 |
| State_South Carolina | 2011 | 1.173207506 | 0.194980938 |
| State_South Carolina | 2012 | 1.264552104 | 0.19530901 |
| State_South Carolina | 2013 | 1.271058984 | 0.196220218 |
| State_South Carolina | 2014 | 1.531874117 | 0.207928657 |
| State_South Carolina | 2015 | 1.651212632 | 0.218400172 |
| State_South Carolina | 2016 | 1.426494648 | 0.196960982 |
| State_South Carolina | 2017 | 1.361869801 | 0.183521714 |
| State_South Carolina | 2018 | 2.078639184 | 0.224934622 |
| State_South Carolina | 2019 | 1.949597123 | 0.220694507 |
| State_South Carolina | 2020 | 2.20151403 | 0.226384273 |
| State_South Carolina | 2021 | 2.542898563 | 0.249704368 |
| State_South Carolina | 2022 | 2.865589134 | 0.260757979 |
| State_South Carolina | 2023 | 1.928349277 | 0.212451418 |
| State_South Dakota | 1999 | NA | NA |
| State_South Dakota | 2000 | NA | 0.564264212 |
| State_South Dakota | 2001 | NA | NA |
| State_South Dakota | 2002 | NA | NA |
| State_South Dakota | 2003 | NA | NA |
| State_South Dakota | 2004 | NA | NA |
| State_South Dakota | 2005 | NA | NA |
| State_South Dakota | 2006 | NA | NA |
| State_South Dakota | 2007 | NA | NA |
| State_South Dakota | 2008 | NA | NA |
| State_South Dakota | 2009 | NA | NA |
| State_South Dakota | 2010 | NA | 0.497669342 |
| State_South Dakota | 2011 | NA | NA |
| State_South Dakota | 2012 | NA | NA |
| State_South Dakota | 2013 | NA | NA |
| State_South Dakota | 2014 | NA | 0.501741533 |
| State_South Dakota | 2015 | NA | 0.5177314 |
| State_South Dakota | 2016 | NA | NA |
| State_South Dakota | 2017 | NA | 0.620670338 |
| State_South Dakota | 2018 | NA | NA |
| State_South Dakota | 2019 | NA | 0.493987721 |
| State_South Dakota | 2020 | 2.598964979 | 0.574480005 |
| State_South Dakota | 2021 | NA | 0.62743874 |
| State_South Dakota | 2022 | NA | 0.537922192 |
| State_South Dakota | 2023 | NA | 0.632310712 |
| State_Tennessee | 1999 | 1.478361473 | 0.205231132 |
| State_Tennessee | 2000 | 1.476415624 | 0.203189132 |
| State_Tennessee | 2001 | 1.444118841 | 0.198879849 |
| State_Tennessee | 2002 | 1.39995326 | 0.194695327 |
| State_Tennessee | 2003 | 1.285698762 | 0.186321752 |
| State_Tennessee | 2004 | 1.459734652 | 0.19766032 |
| State_Tennessee | 2005 | 1.326939321 | 0.18652315 |
| State_Tennessee | 2006 | 1.224072535 | 0.175766373 |
| State_Tennessee | 2007 | 1.309287832 | 0.180478372 |
| State_Tennessee | 2008 | 1.69281706 | 0.201208403 |
| State_Tennessee | 2009 | 1.16990123 | 0.165417802 |
| State_Tennessee | 2010 | 1.401844517 | 0.180647881 |
| State_Tennessee | 2011 | 1.287071494 | 0.171921545 |
| State_Tennessee | 2012 | 0.952777865 | 0.146741173 |
| State_Tennessee | 2013 | 1.403999557 | 0.174500832 |
| State_Tennessee | 2014 | 1.228621512 | 0.161842038 |
| State_Tennessee | 2015 | 1.051232787 | 0.143314855 |
| State_Tennessee | 2016 | 1.092151684 | 0.149060956 |
| State_Tennessee | 2017 | 1.19793302 | 0.155817373 |
| State_Tennessee | 2018 | 1.174669757 | 0.151441961 |
| State_Tennessee | 2019 | 1.511484385 | 0.167623362 |
| State_Tennessee | 2020 | 1.637406386 | 0.172936053 |
| State_Tennessee | 2021 | 2.213020374 | 0.211179456 |
| State_Tennessee | 2022 | 1.812477234 | 0.181913946 |
| State_Tennessee | 2023 | 1.766150531 | 0.179693919 |
| State_Texas | 1999 | 1.136668703 | 0.104317735 |
| State_Texas | 2000 | 1.071199302 | 0.10119154 |
| State_Texas | 2001 | 1.047190142 | 0.099341272 |
| State_Texas | 2002 | 1.029448482 | 0.097745585 |
| State_Texas | 2003 | 1.13236992 | 0.100234609 |
| State_Texas | 2004 | 1.130949127 | 0.100549773 |
| State_Texas | 2005 | 0.877668283 | 0.087289807 |
| State_Texas | 2006 | 1.072318585 | 0.093435758 |
| State_Texas | 2007 | 0.867067284 | 0.084064433 |
| State_Texas | 2008 | 1.012744225 | 0.089956745 |
| State_Texas | 2009 | 0.981108191 | 0.086013226 |
| State_Texas | 2010 | 0.991251284 | 0.085401675 |
| State_Texas | 2011 | 1.042418494 | 0.086689728 |
| State_Texas | 2012 | 0.95969031 | 0.082866708 |
| State_Texas | 2013 | 0.912967527 | 0.078129507 |
| State_Texas | 2014 | 0.942888341 | 0.077624816 |
| State_Texas | 2015 | 1.16799643 | 0.086087706 |
| State_Texas | 2016 | 1.055833997 | 0.080066894 |
| State_Texas | 2017 | 1.18558863 | 0.082824468 |
| State_Texas | 2018 | 1.160487064 | 0.082935536 |
| State_Texas | 2019 | 1.230961998 | 0.083398472 |
| State_Texas | 2020 | 1.499886409 | 0.088954779 |
| State_Texas | 2021 | 1.790223226 | 0.10004371 |
| State_Texas | 2022 | 1.64358702 | 0.092999254 |
| State_Texas | 2023 | 1.68471277 | 0.09378127 |
| State_Utah | 1999 | NA | 0.343292786 |
| State_Utah | 2000 | NA | 0.388093531 |
| State_Utah | 2001 | NA | NA |
| State_Utah | 2002 | NA | NA |
| State_Utah | 2003 | NA | NA |
| State_Utah | 2004 | NA | NA |
| State_Utah | 2005 | NA | 0.284415258 |
| State_Utah | 2006 | NA | NA |
| State_Utah | 2007 | NA | 0.310824677 |
| State_Utah | 2008 | NA | NA |
| State_Utah | 2009 | NA | 0.262041968 |
| State_Utah | 2010 | NA | 0.309198655 |
| State_Utah | 2011 | NA | NA |
| State_Utah | 2012 | NA | 0.28898982 |
| State_Utah | 2013 | NA | 0.254293421 |
| State_Utah | 2014 | NA | 0.295929938 |
| State_Utah | 2015 | 1.495931135 | 0.313946605 |
| State_Utah | 2016 | 1.876314108 | 0.346687971 |
| State_Utah | 2017 | NA | 0.206271608 |
| State_Utah | 2018 | NA | 0.255994968 |
| State_Utah | 2019 | 1.306876805 | 0.270543984 |
| State_Utah | 2020 | 1.563537111 | 0.298498521 |
| State_Utah | 2021 | 1.770405948 | 0.310462118 |
| State_Utah | 2022 | 1.77664213 | 0.312797002 |
| State_Utah | 2023 | 1.200989339 | 0.255772732 |
| State_Vermont | 1999 | NA | NA |
| State_Vermont | 2000 | NA | NA |
| State_Vermont | 2001 | NA | NA |
| State_Vermont | 2002 | NA | NA |
| State_Vermont | 2003 | NA | NA |
| State_Vermont | 2004 | NA | NA |
| State_Vermont | 2005 | NA | NA |
| State_Vermont | 2006 | NA | NA |
| State_Vermont | 2007 | NA | NA |
| State_Vermont | 2008 | NA | NA |
| State_Vermont | 2009 | NA | NA |
| State_Vermont | 2010 | NA | NA |
| State_Vermont | 2011 | NA | NA |
| State_Vermont | 2012 | NA | NA |
| State_Vermont | 2013 | NA | 0.732871638 |
| State_Vermont | 2014 | NA | 0.549439238 |
| State_Vermont | 2015 | NA | NA |
| State_Vermont | 2016 | NA | NA |
| State_Vermont | 2017 | NA | NA |
| State_Vermont | 2018 | NA | NA |
| State_Vermont | 2019 | NA | 0.648509693 |
| State_Vermont | 2020 | NA | 0.652387698 |
| State_Vermont | 2021 | NA | 0.60075713 |
| State_Vermont | 2022 | NA | 0.630749636 |
| State_Vermont | 2023 | NA | 0.558194664 |
| State_Virginia | 1999 | 0.919725478 | 0.153958658 |
| State_Virginia | 2000 | 1.22842564 | 0.174551296 |
| State_Virginia | 2001 | 0.929696497 | 0.149201517 |
| State_Virginia | 2002 | 1.06713113 | 0.15808474 |
| State_Virginia | 2003 | 1.151219636 | 0.163593011 |
| State_Virginia | 2004 | 0.836786048 | 0.136568259 |
| State_Virginia | 2005 | 1.207370868 | 0.165276463 |
| State_Virginia | 2006 | 1.057031545 | 0.15197167 |
| State_Virginia | 2007 | 1.310495352 | 0.168036968 |
| State_Virginia | 2008 | 0.879631731 | 0.135141802 |
| State_Virginia | 2009 | 0.892073297 | 0.134319814 |
| State_Virginia | 2010 | 0.916812169 | 0.134505655 |
| State_Virginia | 2011 | 0.814648008 | 0.127379248 |
| State_Virginia | 2012 | 0.576240099 | 0.102873882 |
| State_Virginia | 2013 | 0.668612301 | 0.111131094 |
| State_Virginia | 2014 | 0.70350816 | 0.111068062 |
| State_Virginia | 2015 | 0.645527124 | 0.105532818 |
| State_Virginia | 2016 | 0.784228083 | 0.116276502 |
| State_Virginia | 2017 | 0.933159285 | 0.124421287 |
| State_Virginia | 2018 | 0.823629893 | 0.114955139 |
| State_Virginia | 2019 | 0.894749582 | 0.121173548 |
| State_Virginia | 2020 | 1.18708361 | 0.133613103 |
| State_Virginia | 2021 | 1.341375182 | 0.143149736 |
| State_Virginia | 2022 | 1.63828486 | 0.155150632 |
| State_Virginia | 2023 | 1.489214506 | 0.148177723 |
| State_Washington | 1999 | 0.787497725 | 0.151646609 |
| State_Washington | 2000 | 0.840661721 | 0.153938321 |
| State_Washington | 2001 | 0.993709201 | 0.166347802 |
| State_Washington | 2002 | 1.032856129 | 0.168043289 |
| State_Washington | 2003 | 0.942601701 | 0.157997225 |
| State_Washington | 2004 | 1.153188604 | 0.174678756 |
| State_Washington | 2005 | 1.057862487 | 0.164393255 |
| State_Washington | 2006 | 1.093107562 | 0.165753815 |
| State_Washington | 2007 | 0.9540854 | 0.155837496 |
| State_Washington | 2008 | 1.228010749 | 0.170346163 |
| State_Washington | 2009 | 0.990344684 | 0.1514741 |
| State_Washington | 2010 | 1.015427578 | 0.149212413 |
| State_Washington | 2011 | 1.15926548 | 0.163119559 |
| State_Washington | 2012 | 0.956548887 | 0.143522204 |
| State_Washington | 2013 | 1.211873266 | 0.163243652 |
| State_Washington | 2014 | 1.269289369 | 0.162595005 |
| State_Washington | 2015 | 1.404726639 | 0.167137566 |
| State_Washington | 2016 | 1.38773137 | 0.16370905 |
| State_Washington | 2017 | 1.469345062 | 0.168442152 |
| State_Washington | 2018 | 1.675380617 | 0.173769911 |
| State_Washington | 2019 | 1.582724006 | 0.168661878 |
| State_Washington | 2020 | 1.940230955 | 0.186395538 |
| State_Washington | 2021 | 2.762796093 | 0.223272828 |
| State_Washington | 2022 | 2.296569633 | 0.19900504 |
| State_Washington | 2023 | 1.763112225 | 0.171674048 |
| State_West Virginia | 1999 | 1.53667645 | 0.335568704 |
| State_West Virginia | 2000 | 1.844006575 | 0.369142273 |
| State_West Virginia | 2001 | NA | 0.277182126 |
| State_West Virginia | 2002 | NA | 0.282905472 |
| State_West Virginia | 2003 | NA | 0.255603679 |
| State_West Virginia | 2004 | NA | 0.275676892 |
| State_West Virginia | 2005 | NA | 0.282781988 |
| State_West Virginia | 2006 | 1.628118402 | 0.333485424 |
| State_West Virginia | 2007 | 1.383250517 | 0.311679614 |
| State_West Virginia | 2008 | NA | 0.298816474 |
| State_West Virginia | 2009 | NA | 0.285859073 |
| State_West Virginia | 2010 | NA | 0.291891948 |
| State_West Virginia | 2011 | NA | 0.251789492 |
| State_West Virginia | 2012 | 1.391520128 | 0.305147419 |
| State_West Virginia | 2013 | NA | 0.279769498 |
| State_West Virginia | 2014 | 1.646781212 | 0.346767128 |
| State_West Virginia | 2015 | NA | 0.227477807 |
| State_West Virginia | 2016 | NA | 0.268442589 |
| State_West Virginia | 2017 | 1.161447427 | 0.26333951 |
| State_West Virginia | 2018 | NA | 0.284480744 |
| State_West Virginia | 2019 | NA | 0.256124519 |
| State_West Virginia | 2020 | NA | 0.228974899 |
| State_West Virginia | 2021 | 1.231304512 | 0.282191285 |
| State_West Virginia | 2022 | 2.027477263 | 0.367182835 |
| State_West Virginia | 2023 | 1.630490938 | 0.305820458 |
| State_Wisconsin | 1999 | 1.000085089 | 0.164788725 |
| State_Wisconsin | 2000 | 1.118137772 | 0.175110007 |
| State_Wisconsin | 2001 | 1.274663531 | 0.186230894 |
| State_Wisconsin | 2002 | 0.877084782 | 0.153104743 |
| State_Wisconsin | 2003 | 0.637384328 | 0.130408814 |
| State_Wisconsin | 2004 | 1.072273108 | 0.168072475 |
| State_Wisconsin | 2005 | 0.88527287 | 0.148302938 |
| State_Wisconsin | 2006 | 0.830222103 | 0.14518441 |
| State_Wisconsin | 2007 | 0.619889649 | 0.124788474 |
| State_Wisconsin | 2008 | 0.70757619 | 0.130301995 |
| State_Wisconsin | 2009 | 0.797944272 | 0.13805733 |
| State_Wisconsin | 2010 | 0.800314755 | 0.141901872 |
| State_Wisconsin | 2011 | 0.878710579 | 0.145810299 |
| State_Wisconsin | 2012 | 0.797088178 | 0.13402518 |
| State_Wisconsin | 2013 | 0.841293792 | 0.139933033 |
| State_Wisconsin | 2014 | 1.307946495 | 0.173488758 |
| State_Wisconsin | 2015 | 1.066648003 | 0.153692051 |
| State_Wisconsin | 2016 | 1.055214911 | 0.146698739 |
| State_Wisconsin | 2017 | 1.526760932 | 0.185716025 |
| State_Wisconsin | 2018 | 1.327780064 | 0.173044321 |
| State_Wisconsin | 2019 | 1.833155027 | 0.19158106 |
| State_Wisconsin | 2020 | 1.906817436 | 0.198430086 |
| State_Wisconsin | 2021 | 2.020212646 | 0.206576685 |
| State_Wisconsin | 2022 | 1.972808167 | 0.195062017 |
| State_Wisconsin | 2023 | 1.698189383 | 0.186843944 |
| State_Wyoming | 1999 | NA | NA |
| State_Wyoming | 2000 | NA | NA |
| State_Wyoming | 2001 | NA | NA |
| State_Wyoming | 2002 | NA | NA |
| State_Wyoming | 2003 | NA | NA |
| State_Wyoming | 2004 | NA | NA |
| State_Wyoming | 2005 | NA | 0 |
| State_Wyoming | 2006 | NA | NA |
| State_Wyoming | 2007 | NA | NA |
| State_Wyoming | 2008 | NA | NA |
| State_Wyoming | 2009 | NA | NA |
| State_Wyoming | 2010 | NA | NA |
| State_Wyoming | 2011 | NA | NA |
| State_Wyoming | 2012 | NA | NA |
| State_Wyoming | 2013 | NA | NA |
| State_Wyoming | 2014 | NA | NA |
| State_Wyoming | 2015 | NA | 0 |
| State_Wyoming | 2016 | NA | NA |
| State_Wyoming | 2017 | NA | NA |
| State_Wyoming | 2018 | NA | NA |
| State_Wyoming | 2019 | NA | 0.826395544 |
| State_Wyoming | 2020 | NA | 0.90090846 |
| State_Wyoming | 2021 | NA | 0.905793812 |
| State_Wyoming | 2022 | NA | 0.800014517 |
| State_Wyoming | 2023 | NA | NA |
| Urbanization_Metropolitan | 1999 | 0.975513754 | 0.026037162 |
| Urbanization_Metropolitan | 2000 | 0.955493664 | 0.025642582 |
| Urbanization_Metropolitan | 2001 | 0.938350587 | 0.025450583 |
| Urbanization_Metropolitan | 2002 | 0.918020649 | 0.024711208 |
| Urbanization_Metropolitan | 2003 | 0.917924025 | 0.024573459 |
| Urbanization_Metropolitan | 2004 | 0.909589389 | 0.024322504 |
| Urbanization_Metropolitan | 2005 | 0.874132521 | 0.023463672 |
| Urbanization_Metropolitan | 2006 | 0.893158696 | 0.023575984 |
| Urbanization_Metropolitan | 2007 | 0.85511328 | 0.022756116 |
| Urbanization_Metropolitan | 2008 | 0.87163763 | 0.023159704 |
| Urbanization_Metropolitan | 2009 | 0.838864525 | 0.022104898 |
| Urbanization_Metropolitan | 2010 | 0.882471122 | 0.022809125 |
| Urbanization_Metropolitan | 2011 | 0.857511547 | 0.022077246 |
| Urbanization_Metropolitan | 2012 | 0.841505335 | 0.021596282 |
| Urbanization_Metropolitan | 2013 | 0.818404912 | 0.021100095 |
| Urbanization_Metropolitan | 2014 | 0.840395789 | 0.021034474 |
| Urbanization_Metropolitan | 2015 | 0.947112504 | 0.022354961 |
| Urbanization_Metropolitan | 2016 | 0.892830809 | 0.021000455 |
| Urbanization_Metropolitan | 2017 | 0.989748672 | 0.022482636 |
| Urbanization_Metropolitan | 2018 | 1.040564576 | 0.022377685 |
| Urbanization_Metropolitan | 2019 | 1.067448613 | 0.022343876 |
| Urbanization_Metropolitan | 2020 | 1.379667706 | 0.025282984 |
| Urbanization_Nonmetropolitan | 1999 | 1.109233273 | 0.057568487 |
| Urbanization_Nonmetropolitan | 2000 | 1.063334027 | 0.055915388 |
| Urbanization_Nonmetropolitan | 2001 | 0.927735655 | 0.052880817 |
| Urbanization_Nonmetropolitan | 2002 | 1.04248116 | 0.055884893 |
| Urbanization_Nonmetropolitan | 2003 | 1.111701812 | 0.057286301 |
| Urbanization_Nonmetropolitan | 2004 | 1.144821958 | 0.058592515 |
| Urbanization_Nonmetropolitan | 2005 | 0.955514642 | 0.053180297 |
| Urbanization_Nonmetropolitan | 2006 | 1.007024393 | 0.053033177 |
| Urbanization_Nonmetropolitan | 2007 | 0.929749462 | 0.050810755 |
| Urbanization_Nonmetropolitan | 2008 | 1.041265847 | 0.053753814 |
| Urbanization_Nonmetropolitan | 2009 | 1.074276298 | 0.055640043 |
| Urbanization_Nonmetropolitan | 2010 | 0.97114233 | 0.050738102 |
| Urbanization_Nonmetropolitan | 2011 | 0.988165115 | 0.051513107 |
| Urbanization_Nonmetropolitan | 2012 | 0.927893111 | 0.04945114 |
| Urbanization_Nonmetropolitan | 2013 | 0.968462679 | 0.05035615 |
| Urbanization_Nonmetropolitan | 2014 | 1.035983802 | 0.052384321 |
| Urbanization_Nonmetropolitan | 2015 | 1.071129627 | 0.054086914 |
| Urbanization_Nonmetropolitan | 2016 | 1.075547177 | 0.052740093 |
| Urbanization_Nonmetropolitan | 2017 | 1.121143599 | 0.053164804 |
| Urbanization_Nonmetropolitan | 2018 | 1.12209099 | 0.052748059 |
| Urbanization_Nonmetropolitan | 2019 | 1.348151034 | 0.058732527 |
| Urbanization_Nonmetropolitan | 2020 | 1.489242933 | 0.060136838 |
| Age Groups_25-34 years | 1999 | NA | NA |
| Age Groups_25-34 years | 2000 | NA | 0.009379533 |
| Age Groups_25-34 years | 2001 | NA | 0.010445773 |
| Age Groups_25-34 years | 2002 | NA | 0.009508745 |
| Age Groups_25-34 years | 2003 | NA | 0.009534392 |
| Age Groups_25-34 years | 2004 | NA | 0.010804718 |
| Age Groups_25-34 years | 2005 | NA | NA |
| Age Groups_25-34 years | 2006 | NA | 0.009152265 |
| Age Groups_25-34 years | 2007 | NA | 0.007962735 |
| Age Groups_25-34 years | 2008 | NA | NA |
| Age Groups_25-34 years | 2009 | NA | 0.008853771 |
| Age Groups_25-34 years | 2010 | NA | 0.009740905 |
| Age Groups_25-34 years | 2011 | NA | 0.008953369 |
| Age Groups_25-34 years | 2012 | NA | 0.010027674 |
| Age Groups_25-34 years | 2013 | NA | 0.009623399 |
| Age Groups_25-34 years | 2014 | NA | 0.007960432 |
| Age Groups_25-34 years | 2015 | 0.052110236 | 0.010865735 |
| Age Groups_25-34 years | 2016 | 0.04476552 | 0.010009874 |
| Age Groups_25-34 years | 2017 | 0.044108561 | 0.009862974 |
| Age Groups_25-34 years | 2018 | 0.065648712 | 0.01198576 |
| Age Groups_25-34 years | 2019 | 0.080539272 | 0.013240575 |
| Age Groups_25-34 years | 2020 | 0.067289425 | 0.012085538 |
| Age Groups_25-34 years | 2021 | 0.107703895 | 0.015386271 |
| Age Groups_25-34 years | 2022 | 0.0725254 | 0.012625052 |
| Age Groups_25-34 years | 2023 | 0.074655515 | 0.012803315 |
| Age Groups_35-44 years | 1999 | 0.09317457 | 0.014377148 |
| Age Groups_35-44 years | 2000 | 0.099671026 | 0.014858079 |
| Age Groups_35-44 years | 2001 | 0.082127772 | 0.013501723 |
| Age Groups_35-44 years | 2002 | 0.103045097 | 0.015193171 |
| Age Groups_35-44 years | 2003 | 0.104180336 | 0.015360552 |
| Age Groups_35-44 years | 2004 | 0.111871444 | 0.015981635 |
| Age Groups_35-44 years | 2005 | 0.087345202 | 0.014169263 |
| Age Groups_35-44 years | 2006 | 0.097123747 | 0.01498652 |
| Age Groups_35-44 years | 2007 | 0.130852648 | 0.01748592 |
| Age Groups_35-44 years | 2008 | 0.071102708 | 0.012981519 |
| Age Groups_35-44 years | 2009 | 0.108465592 | 0.016169096 |
| Age Groups_35-44 years | 2010 | 0.082784267 | 0.014197385 |
| Age Groups_35-44 years | 2011 | 0.09599302 | 0.015371185 |
| Age Groups_35-44 years | 2012 | 0.098725406 | 0.015609857 |
| Age Groups_35-44 years | 2013 | 0.096408916 | 0.015437782 |
| Age Groups_35-44 years | 2014 | 0.125885105 | 0.017627441 |
| Age Groups_35-44 years | 2015 | 0.137965754 | 0.018436449 |
| Age Groups_35-44 years | 2016 | 0.121076875 | 0.017296696 |
| Age Groups_35-44 years | 2017 | 0.163912889 | 0.020025147 |
| Age Groups_35-44 years | 2018 | 0.135665856 | 0.018129113 |
| Age Groups_35-44 years | 2019 | 0.153627737 | 0.019203467 |
| Age Groups_35-44 years | 2020 | 0.204100076 | 0.022008677 |
| Age Groups_35-44 years | 2021 | 0.26495343 | 0.024707035 |
| Age Groups_35-44 years | 2022 | 0.189951497 | 0.020849885 |
| Age Groups_35-44 years | 2023 | 0.220766997 | 0.022300834 |
| Age Groups_45-54 years | 1999 | 0.205042296 | 0.023676245 |
| Age Groups_45-54 years | 2000 | 0.273369423 | 0.02693589 |
| Age Groups_45-54 years | 2001 | 0.269129332 | 0.026140152 |
| Age Groups_45-54 years | 2002 | 0.19753855 | 0.022224823 |
| Age Groups_45-54 years | 2003 | 0.284174745 | 0.026384963 |
| Age Groups_45-54 years | 2004 | 0.27384144 | 0.025647601 |
| Age Groups_45-54 years | 2005 | 0.240023133 | 0.023765832 |
| Age Groups_45-54 years | 2006 | 0.258743216 | 0.024448936 |
| Age Groups_45-54 years | 2007 | 0.277651728 | 0.025137406 |
| Age Groups_45-54 years | 2008 | 0.287896341 | 0.025446682 |
| Age Groups_45-54 years | 2009 | 0.314261536 | 0.026465596 |
| Age Groups_45-54 years | 2010 | 0.306620905 | 0.026101305 |
| Age Groups_45-54 years | 2011 | 0.337670098 | 0.027479203 |
| Age Groups_45-54 years | 2012 | 0.30721454 | 0.02634343 |
| Age Groups_45-54 years | 2013 | 0.313017421 | 0.026742883 |
| Age Groups_45-54 years | 2014 | 0.370465386 | 0.029196763 |
| Age Groups_45-54 years | 2015 | 0.368156449 | 0.029196706 |
| Age Groups_45-54 years | 2016 | 0.33421617 | 0.02794856 |
| Age Groups_45-54 years | 2017 | 0.377581549 | 0.029850442 |
| Age Groups_45-54 years | 2018 | 0.405940675 | 0.031226206 |
| Age Groups_45-54 years | 2019 | 0.440368028 | 0.032823095 |
| Age Groups_45-54 years | 2020 | 0.532624713 | 0.036324704 |
| Age Groups_45-54 years | 2021 | 0.754514133 | 0.043062396 |
| Age Groups_45-54 years | 2022 | 0.571334656 | 0.037591061 |
| Age Groups_45-54 years | 2023 | 0.521067667 | 0.035871778 |
| Age Groups_55-64 years | 1999 | 0.841112715 | 0.05947565 |
| Age Groups_55-64 years | 2000 | 0.741513257 | 0.055269135 |
| Age Groups_55-64 years | 2001 | 0.784695022 | 0.055907205 |
| Age Groups_55-64 years | 2002 | 0.763949607 | 0.053487171 |
| Age Groups_55-64 years | 2003 | 0.72833875 | 0.050993912 |
| Age Groups_55-64 years | 2004 | 0.706356774 | 0.04909519 |
| Age Groups_55-64 years | 2005 | 0.675554461 | 0.046954281 |
| Age Groups_55-64 years | 2006 | 0.7860918 | 0.049617674 |
| Age Groups_55-64 years | 2007 | 0.697286204 | 0.045878064 |
| Age Groups_55-64 years | 2008 | 0.600168697 | 0.0419176 |
| Age Groups_55-64 years | 2009 | 0.700454165 | 0.044478884 |
| Age Groups_55-64 years | 2010 | 0.677032686 | 0.043078558 |
| Age Groups_55-64 years | 2011 | 0.701484467 | 0.042930152 |
| Age Groups_55-64 years | 2012 | 0.689365592 | 0.042267716 |
| Age Groups_55-64 years | 2013 | 0.778300553 | 0.044492481 |
| Age Groups_55-64 years | 2014 | 0.803441705 | 0.044774056 |
| Age Groups_55-64 years | 2015 | 0.87088795 | 0.046156969 |
| Age Groups_55-64 years | 2016 | 0.899594107 | 0.046579217 |
| Age Groups_55-64 years | 2017 | 0.988197399 | 0.048508703 |
| Age Groups_55-64 years | 2018 | 1.014840901 | 0.04899698 |
| Age Groups_55-64 years | 2019 | 1.083665145 | 0.050526148 |
| Age Groups_55-64 years | 2020 | 1.556468794 | 0.060585466 |
| Age Groups_55-64 years | 2021 | 1.747538447 | 0.063896334 |
| Age Groups_55-64 years | 2022 | 1.549229488 | 0.060672509 |
| Age Groups_55-64 years | 2023 | 1.560170086 | 0.061054173 |
| Age Groups_65-74 years | 1999 | 2.128247661 | 0.10749274 |
| Age Groups_65-74 years | 2000 | 2.196728332 | 0.109291319 |
| Age Groups_65-74 years | 2001 | 1.914689799 | 0.102053309 |
| Age Groups_65-74 years | 2002 | 2.050190513 | 0.105590183 |
| Age Groups_65-74 years | 2003 | 1.891798325 | 0.101120874 |
| Age Groups_65-74 years | 2004 | 1.901697455 | 0.100931621 |
| Age Groups_65-74 years | 2005 | 1.911904423 | 0.100626549 |
| Age Groups_65-74 years | 2006 | 1.843459367 | 0.09797876 |
| Age Groups_65-74 years | 2007 | 1.80214691 | 0.095648027 |
| Age Groups_65-74 years | 2008 | 1.911665544 | 0.096553691 |
| Age Groups_65-74 years | 2009 | 1.681337237 | 0.088985803 |
| Age Groups_65-74 years | 2010 | 1.832967055 | 0.091878336 |
| Age Groups_65-74 years | 2011 | 1.752533545 | 0.088291364 |
| Age Groups_65-74 years | 2012 | 1.730219794 | 0.084933149 |
| Age Groups_65-74 years | 2013 | 1.673489773 | 0.081464207 |
| Age Groups_65-74 years | 2014 | 1.803147098 | 0.082647112 |
| Age Groups_65-74 years | 2015 | 1.912849766 | 0.083325045 |
| Age Groups_65-74 years | 2016 | 1.847690893 | 0.080334387 |
| Age Groups_65-74 years | 2017 | 1.980902083 | 0.081691025 |
| Age Groups_65-74 years | 2018 | 2.069373806 | 0.08238047 |
| Age Groups_65-74 years | 2019 | 2.220215311 | 0.083976255 |
| Age Groups_65-74 years | 2020 | 2.841834433 | 0.093438941 |
| Age Groups_65-74 years | 2021 | 3.540651341 | 0.102552213 |
| Age Groups_65-74 years | 2022 | 3.252591811 | 0.09811394 |
| Age Groups_65-74 years | 2023 | 3.188672176 | 0.095880946 |
| Age Groups_75-84 years | 1999 | 5.243390669 | 0.20710148 |
| Age Groups_75-84 years | 2000 | 5.137050023 | 0.203857612 |
| Age Groups_75-84 years | 2001 | 4.819901636 | 0.195633772 |
| Age Groups_75-84 years | 2002 | 4.755240635 | 0.193009264 |
| Age Groups_75-84 years | 2003 | 4.962610606 | 0.196164408 |
| Age Groups_75-84 years | 2004 | 5.019283054 | 0.196570295 |
| Age Groups_75-84 years | 2005 | 4.458958537 | 0.184671194 |
| Age Groups_75-84 years | 2006 | 4.734576944 | 0.190145186 |
| Age Groups_75-84 years | 2007 | 4.508139446 | 0.185597164 |
| Age Groups_75-84 years | 2008 | 4.634408633 | 0.188259982 |
| Age Groups_75-84 years | 2009 | 4.438378149 | 0.184612193 |
| Age Groups_75-84 years | 2010 | 4.685661768 | 0.189406651 |
| Age Groups_75-84 years | 2011 | 4.432560191 | 0.183420646 |
| Age Groups_75-84 years | 2012 | 4.226742032 | 0.178453188 |
| Age Groups_75-84 years | 2013 | 4.082841068 | 0.174251411 |
| Age Groups_75-84 years | 2014 | 4.121996479 | 0.173567363 |
| Age Groups_75-84 years | 2015 | 4.690022548 | 0.183534765 |
| Age Groups_75-84 years | 2016 | 4.355910486 | 0.174937575 |
| Age Groups_75-84 years | 2017 | 4.950174925 | 0.183465689 |
| Age Groups_75-84 years | 2018 | 5.099265485 | 0.18200059 |
| Age Groups_75-84 years | 2019 | 4.990647389 | 0.1767778 |
| Age Groups_75-84 years | 2020 | 5.956886608 | 0.190285763 |
| Age Groups_75-84 years | 2021 | 7.682304321 | 0.217724264 |
| Age Groups_75-84 years | 2022 | 7.300001227 | 0.204120988 |
| Age Groups_75-84 years | 2023 | 6.772612318 | 0.192019806 |
| Age Groups_85+ years | 1999 | 10.27920438 | 0.497445565 |
| Age Groups_85+ years | 2000 | 9.151834837 | 0.46461402 |
| Age Groups_85+ years | 2001 | 8.742041148 | 0.450238026 |
| Age Groups_85+ years | 2002 | 9.522048119 | 0.466856819 |
| Age Groups_85+ years | 2003 | 9.471189671 | 0.460505 |
| Age Groups_85+ years | 2004 | 9.107141561 | 0.447591589 |
| Age Groups_85+ years | 2005 | 9.289840686 | 0.44490268 |
| Age Groups_85+ years | 2006 | 8.980813325 | 0.429610563 |
| Age Groups_85+ years | 2007 | 8.115812043 | 0.401301078 |
| Age Groups_85+ years | 2008 | 8.814744103 | 0.411885943 |
| Age Groups_85+ years | 2009 | 9.07346169 | 0.411157796 |
| Age Groups_85+ years | 2010 | 8.373634483 | 0.390422721 |
| Age Groups_85+ years | 2011 | 8.54079178 | 0.385833643 |
| Age Groups_85+ years | 2012 | 8.339943574 | 0.376376385 |
| Age Groups_85+ years | 2013 | 7.697669957 | 0.356970897 |
| Age Groups_85+ years | 2014 | 7.659563557 | 0.35255999 |
| Age Groups_85+ years | 2015 | 8.334445388 | 0.364091937 |
| Age Groups_85+ years | 2016 | 9.012071631 | 0.375829367 |
| Age Groups_85+ years | 2017 | 8.239700143 | 0.356901031 |
| Age Groups_85+ years | 2018 | 9.229119461 | 0.375527545 |
| Age Groups_85+ years | 2019 | 10.11361465 | 0.391307499 |
| Age Groups_85+ years | 2020 | 12.69069839 | 0.436573297 |
| Age Groups_85+ years | 2021 | 15.02738733 | 0.50147041 |
| Age Groups_85+ years | 2022 | 15.6339907 | 0.490965383 |
| Age Groups_85+ years | 2023 | 14.9798708 | 0.49173835 |

Supplementary table 3. Characteristics with APC, 95% CI, and Time Range.

| Metric | Measure | Start | End | APC | lower | upper | P value |
| --- | --- | --- | --- | --- | --- | --- | --- |
| Census Region | Midwest | 1999 | 2012 | -1.4106 | -1.9704 | -0.8476 | 0.000102 |
| Census Region | Midwest | 2012 | 2018 | 3.5834 | 1.2359 | 5.9854 | 0.005325 |
| Census Region | Midwest | 2018 | 2021 | 17.8299 | 8.202 | 28.3146 | 0.001024 |
| Census Region | Midwest | 2021 | 2023 | -6.3021 | -13.2084 | 1.1539 | 0.08966 |
| Census Region | Northeast | 1999 | 2017 | -0.228 | -0.8469 | 0.3948 | 0.449578 |
| Census Region | Northeast | 2017 | 2021 | 16.8297 | 8.089 | 26.2771 | 0.000576 |
| Census Region | Northeast | 2021 | 2023 | -2.9091 | -14.9624 | 10.8526 | 0.644397 |
| Census Region | South | 1999 | 2013 | -1.4742 | -2.2026 | -0.7404 | 0.000744 |
| Census Region | South | 2013 | 2018 | 3.3748 | -1.1917 | 8.1523 | 0.137431 |
| Census Region | South | 2018 | 2021 | 18.5854 | 5.3239 | 33.5167 | 0.008103 |
| Census Region | South | 2021 | 2023 | -2.6716 | -12.1043 | 7.7733 | 0.577872 |
| Census Region | West | 1999 | 2017 | 0.3359 | -0.2818 | 0.9574 | 0.267778 |
| Census Region | West | 2017 | 2021 | 16.9315 | 8.8267 | 25.6398 | 0.000258 |
| Census Region | West | 2021 | 2023 | -4.5442 | -15.4773 | 7.8031 | 0.431022 |
| Race | Hispanic | 1999 | 2018 | 0.0263 | -1.0824 | 1.1475 | 0.960876 |
| Race | Hispanic | 2018 | 2021 | 22.6178 | -3.2564 | 55.412 | 0.08719 |
| Race | Hispanic | 2021 | 2023 | -7.3997 | -24.7128 | 13.8947 | 0.444036 |
| Race | NH Black | 1999 | 2017 | -1.5141 | -2.21 | -0.8133 | 0.00029 |
| Race | NH Black | 2017 | 2021 | 21.3315 | 11.6713 | 31.8275 | 0.00013 |
| Race | NH Black | 2021 | 2023 | -7.4895 | -19.8393 | 6.763 | 0.267572 |
| Race | NH White | 1999 | 2013 | -0.6838 | -1.1336 | -0.2321 | 0.005898 |
| Race | NH White | 2013 | 2018 | 3.8051 | 0.9257 | 6.7667 | 0.012918 |
| Race | NH White | 2018 | 2021 | 17.2081 | 8.7124 | 26.3678 | 0.000475 |
| Race | NH White | 2021 | 2023 | -2.7471 | -8.9782 | 3.9105 | 0.382176 |
| Sex | Both | 1999 | 2013 | -1.0089 | -1.4104 | -0.6057 | 0.000102 |
| Sex | Both | 2013 | 2018 | 3.6616 | 1.1113 | 6.2762 | 0.007889 |
| Sex | Both | 2018 | 2021 | 18.5666 | 11.0323 | 26.6123 | 7.00E-05 |
| Sex | Both | 2021 | 2023 | -4.3989 | -9.6773 | 1.1881 | 0.111466 |
| Sex | Female | 1999 | 2013 | -1.1622 | -1.6417 | -0.6804 | 0.000146 |
| Sex | Female | 2013 | 2018 | 3.8289 | 0.7106 | 7.0438 | 0.019299 |
| Sex | Female | 2018 | 2021 | 17.144 | 8.0442 | 27.0102 | 0.000896 |
| Sex | Female | 2021 | 2023 | -2.21 | -8.7973 | 4.853 | 0.503111 |
| Sex | Male | 1999 | 2012 | -1.0077 | -1.6943 | -0.3163 | 0.007513 |
| Sex | Male | 2012 | 2018 | 2.2707 | -0.3744 | 4.9861 | 0.08741 |
| Sex | Male | 2018 | 2021 | 21.1938 | 10.1171 | 33.3847 | 0.000732 |
| Sex | Male | 2021 | 2023 | -6.3698 | -13.838 | 1.7456 | 0.111565 |
| State | Alabama | 1999 | 2014 | -2.6624 | -4.9382 | -0.3322 | 0.027413 |
| State | Alabama | 2014 | 2023 | 5.0035 | 0.5066 | 9.7015 | 0.030597 |
| State | California | 1999 | 2014 | -0.8214 | -2.0355 | 0.4077 | 0.177749 |
| State | California | 2014 | 2023 | 5.6107 | 3.4169 | 7.8509 | 2.60E-05 |
| State | Florida | 1999 | 2013 | -0.4417 | -2.4823 | 1.6417 | 0.660485 |
| State | Florida | 2013 | 2023 | 8.6881 | 6.0129 | 11.4308 | 1.00E-06 |
| State | Georgia | 1999 | 2016 | -1.6624 | -3.42 | 0.1271 | 0.066738 |
| State | Georgia | 2016 | 2023 | 9.5685 | 3.8255 | 15.6291 | 0.002053 |
| State | Illinois | 1999 | 2012 | -3.5654 | -5.3923 | -1.7032 | 0.000773 |
| State | Illinois | 2012 | 2023 | 3.9538 | 1.5765 | 6.3867 | 0.002274 |
| State | Indiana | 1999 | 2023 | 2.5361 | 1.4022 | 3.6828 | 0.000109 |
| State | Maryland | 1999 | 2017 | -1.9521 | -3.4597 | -0.4211 | 0.015678 |
| State | Maryland | 2017 | 2020 | 43.6717 | -0.3942 | 107.2324 | 0.05225 |
| State | Maryland | 2020 | 2023 | -3.5532 | -15.6925 | 10.3341 | 0.577849 |
| State | Massachusetts | 1999 | 2015 | -0.4219 | -3.0772 | 2.3061 | 0.747549 |
| State | Massachusetts | 2015 | 2023 | 10.5171 | 4.5084 | 16.8713 | 0.001317 |
| State | Michigan | 1999 | 2016 | -0.5751 | -2.065 | 0.9374 | 0.434887 |
| State | Michigan | 2016 | 2023 | 12.0516 | 7.258 | 17.0594 | 2.60E-05 |
| State | Minnesota | 1999 | 2012 | -0.0878 | -3.0353 | 2.9493 | 0.951826 |
| State | Minnesota | 2012 | 2023 | 12.1297 | 9.4156 | 14.9111 | 0 |
| State | Missouri | 1999 | 2015 | -2.1922 | -3.6869 | -0.6743 | 0.007039 |
| State | Missouri | 2015 | 2023 | 8.5621 | 4.796 | 12.4635 | 9.60E-05 |
| State | New Jersey | 1999 | 2016 | 0.4153 | -1.4007 | 2.2647 | 0.640844 |
| State | New Jersey | 2016 | 2023 | 8.201 | 2.4746 | 14.2474 | 0.006708 |
| State | New York | 1999 | 2016 | -0.9309 | -1.7248 | -0.1305 | 0.025304 |
| State | New York | 2016 | 2020 | 18.3073 | 7.0651 | 30.73 | 0.00245 |
| State | New York | 2020 | 2023 | 0.6197 | -6.784 | 8.6114 | 0.866599 |
| State | North Carolina | 1999 | 2018 | -2.1507 | -3.4959 | -0.7868 | 0.003777 |
| State | North Carolina | 2018 | 2023 | 16.2008 | 6.8019 | 26.4268 | 0.001374 |
| State | Ohio | 1999 | 2018 | -1.7902 | -2.7075 | -0.8642 | 0.000812 |
| State | Ohio | 2018 | 2021 | 26.3868 | -5.0727 | 68.272 | 0.102457 |
| State | Ohio | 2021 | 2023 | -13.3101 | -32.5789 | 11.4658 | 0.247057 |
| State | Pennsylvania | 1999 | 2017 | -0.6058 | -1.7735 | 0.5759 | 0.293212 |
| State | Pennsylvania | 2017 | 2021 | 20.1925 | 3.2453 | 39.9216 | 0.020576 |
| State | Pennsylvania | 2021 | 2023 | -5.9791 | -27.3582 | 21.6921 | 0.620581 |
| State | South Carolina | 1999 | 2011 | -2.1342 | -5.1793 | 1.0087 | 0.169966 |
| State | South Carolina | 2011 | 2023 | 6.7526 | 4.2138 | 9.3532 | 1.50E-05 |
| State | Tennessee | 1999 | 2018 | -1.3243 | -2.32 | -0.3186 | 0.013007 |
| State | Tennessee | 2018 | 2021 | 22.6949 | -6.5687 | 61.124 | 0.131684 |
| State | Tennessee | 2021 | 2023 | -10.1477 | -30.3216 | 15.8671 | 0.387016 |
| State | Texas | 1999 | 2013 | -1.2448 | -2.5355 | 0.0629 | 0.060882 |
| State | Texas | 2013 | 2023 | 6.6265 | 4.9073 | 8.374 | 0 |
| State | Virginia | 1999 | 2015 | -3.4623 | -5.3824 | -1.5032 | 0.001561 |
| State | Virginia | 2015 | 2023 | 11.0098 | 6.286 | 15.9435 | 6.70E-05 |
| State | Washington | 1999 | 2018 | 2.7036 | 1.7946 | 3.6208 | 8.00E-06 |
| State | Washington | 2018 | 2021 | 22.3948 | -0.6271 | 50.7502 | 0.056534 |
| State | Washington | 2021 | 2023 | -16.9189 | -31.2473 | 0.3957 | 0.054437 |
| State | Wisconsin | 1999 | 2008 | -5.3916 | -10.2045 | -0.3207 | 0.038588 |
| State | Wisconsin | 2008 | 2023 | 7.6828 | 5.6502 | 9.7545 | 0 |
| Urbanization | Metropolitan | 1999 | 2013 | -1.0353 | -1.4403 | -0.6285 | 8.70E-05 |
| Urbanization | Metropolitan | 2013 | 2018 | 4.0141 | 1.4448 | 6.6484 | 0.004533 |
| Urbanization | Metropolitan | 2018 | 2020 | 15.217 | 7.9307 | 22.9953 | 0.000375 |
| Urbanization | Nonmetropolitan | 1999 | 2015 | -0.4679 | -1.1938 | 0.2634 | 0.194194 |
| Urbanization | Nonmetropolitan | 2015 | 2020 | 7.8719 | 4.0121 | 11.8748 | 0.000402 |
| Age Groups | 35-44 years | 1999 | 2012 | 0.1067 | -2.7411 | 3.0379 | 0.939307 |
| Age Groups | 35-44 years | 2012 | 2023 | 8.3794 | 5.2573 | 11.5942 | 1.30E-05 |
| Age Groups | 45-54 years | 1999 | 2018 | 2.6501 | 1.8414 | 3.4652 | 2.00E-06 |
| Age Groups | 45-54 years | 2018 | 2021 | 23.0148 | -0.1125 | 51.4968 | 0.051117 |
| Age Groups | 45-54 years | 2021 | 2023 | -15.2619 | -30.3653 | 3.1174 | 0.092977 |
| Age Groups | 55-64 years | 1999 | 2010 | -1.7607 | -2.9319 | -0.5754 | 0.006725 |
| Age Groups | 55-64 years | 2010 | 2018 | 5.4448 | 3.4439 | 7.4844 | 3.60E-05 |
| Age Groups | 55-64 years | 2018 | 2021 | 20.2117 | 7.5566 | 34.3557 | 0.003205 |
| Age Groups | 55-64 years | 2021 | 2023 | -6.579 | -15.587 | 3.3902 | 0.171979 |
| Age Groups | 65-74 years | 1999 | 2012 | -1.5946 | -2.2258 | -0.9593 | 0.000101 |
| Age Groups | 65-74 years | 2012 | 2018 | 2.5835 | 0.1376 | 5.0892 | 0.039759 |
| Age Groups | 65-74 years | 2018 | 2021 | 20.176 | 10.3382 | 30.8908 | 0.000401 |
| Age Groups | 65-74 years | 2021 | 2023 | -4.2098 | -10.9163 | 3.0014 | 0.224467 |
| Age Groups | 75-84 years | 1999 | 2014 | -1.5124 | -2.509 | -0.5055 | 0.005329 |
| Age Groups | 75-84 years | 2014 | 2023 | 6.762 | 4.8552 | 8.7035 | 0 |
| Age Groups | 85+ years | 1999 | 2017 | -1.0131 | -1.5432 | -0.48 | 0.000927 |
| Age Groups | 85+ years | 2017 | 2021 | 16.8267 | 9.2464 | 24.933 | 0.000138 |
| Age Groups | 85+ years | 2021 | 2023 | 1.3835 | -9.6218 | 13.7288 | 0.80385 |

Supplementary table 4. Characteristics with AAPC and 95% CI.

| Metric | Measure | AAPC | lower | upper | P value |
| --- | --- | --- | --- | --- | --- |
| Census Region | Midwest | 1.6323 | 0.3384 | 2.9429 | 0.013262 |
| Census Region | Northeast | 2.1991 | 0.5364 | 3.8892 | 0.009345 |
| Census Region | South | 1.7453 | -0.0957 | 3.6202 | 0.063277 |
| Census Region | West | 2.5017 | 0.956 | 4.0711 | 0.001436 |
| Race | Hispanic | 1.9477 | -1.3503 | 5.3559 | 0.250277 |
| Race | NH Black | 1.4399 | -0.3375 | 3.2489 | 0.112937 |
| Race | NH White | 2.1532 | 0.9783 | 3.3419 | 0.000307 |
| Sex | Both | 1.93 | 0.9051 | 2.9654 | 0.000209 |
| Sex | Female | 1.9106 | 0.6539 | 3.1829 | 0.002794 |
| Sex | Male | 1.8846 | 0.4269 | 3.3633 | 0.011103 |
| State | Alabama | 0.1444 | -1.9129 | 2.2448 | 0.891628 |
| State | California | 1.5434 | 0.4984 | 2.5993 | 0.00371 |
| State | Florida | 3.2653 | 1.7311 | 4.8226 | 2.60E-05 |
| State | Georgia | 1.4888 | -0.4233 | 3.4375 | 0.127802 |
| State | Illinois | -0.1891 | -1.5697 | 1.2109 | 0.789975 |
| State | Indiana | 2.5361 | 1.4022 | 3.6828 | 0.000109 |
| State | Maryland | 2.6328 | -2.0385 | 7.5267 | 0.274216 |
| State | Massachusetts | 3.0985 | 0.6178 | 5.6403 | 0.014063 |
| State | Michigan | 2.9531 | 1.3556 | 4.5757 | 0.000265 |
| State | Minnesota | 5.3373 | 3.4026 | 7.3081 | 0 |
| State | Missouri | 1.2687 | -0.2065 | 2.7657 | 0.092216 |
| State | New Jersey | 2.6264 | 0.6723 | 4.6184 | 0.008216 |
| State | New York | 2.2415 | 0.3578 | 4.1605 | 0.019471 |
| State | North Carolina | 1.4169 | -0.5374 | 3.4095 | 0.156438 |
| State | Ohio | 0.3074 | -3.5418 | 4.3101 | 0.877835 |
| State | Pennsylvania | 2.1181 | -1.0924 | 5.4328 | 0.198444 |
| State | South Carolina | 2.2126 | 0.3225 | 4.1384 | 0.021557 |
| State | Tennessee | 0.6114 | -3.1404 | 4.5085 | 0.75326 |
| State | Texas | 1.9617 | 0.9858 | 2.947 | 7.50E-05 |
| State | Virginia | 1.139 | -0.7192 | 3.0319 | 0.231294 |
| State | Washington | 3.1417 | 0.1901 | 6.1802 | 0.036783 |
| State | Wisconsin | 2.5806 | 0.3953 | 4.8134 | 0.020392 |
| Urbanization | Metropolitan | 1.605 | 0.7695 | 2.4475 | 0.000157 |
| Urbanization | Nonmetropolitan | 1.4573 | 0.4899 | 2.4341 | 0.003079 |
| Age Groups | 35-44 years | 3.817 | 1.828 | 5.8448 | 0.000147 |
| Age Groups | 45-54 years | 3.3342 | 0.3657 | 6.3905 | 0.027427 |
| Age Groups | 55-64 years | 2.7234 | 1.0177 | 4.4579 | 0.001659 |
| Age Groups | 65-74 years | 1.7205 | 0.4173 | 3.0407 | 0.009515 |
| Age Groups | 75-84 years | 1.5126 | 0.6315 | 2.4014 | 0.000738 |
| Age Groups | 85+ years | 1.9619 | 0.5264 | 3.4178 | 0.007235 |
